# Meta‐analysis of management effects on biodiversity in plantation and secondary forests of Japan

**DOI:** 10.1111/csp2.14

**Published:** 2019-03-20

**Authors:** Rebecca Spake, Sakiko Yanou, Yuichi Yamaura, Kazuhiro Kawamura, Kanehiro Kitayama, C. Patrick Doncaster

**Affiliations:** ^1^ School of Geography and Environmental Science University of Southampton Southampton UK; ^2^ School of Biological Sciences University of Southampton Southampton UK; ^3^ Graduate School of Agriculture, Kyoto University Kyoto Japan; ^4^ Department of Forest Vegetation Forestry and Forest Products Research Institute Ibaraki Japan; ^5^ Fenner School of Environment and Society Australian National University Canberra Australian Capital Territory Australia; ^6^ Graduate School of Agriculture Hokkaido University Hokkaido Japan

**Keywords:** coppice, richness, satoyama, synthesis, thinning, traditional management

## Abstract

Conservation of temperate forest biodiversity has historically focused on natural old‐growth. Less than 3% of the world's temperate forests remain unmodified by humans, however, and much of temperate‐forest biodiversity is held in the predominating planted and secondary forests. Japan provides a widely applicable model for examining how to maximize biodiversity in managed temperate forests, because of its richness of forestry research generated from its vast forest area, albeit largely in Japanese, and the wide practice of its dominant management interventions across the northern temperate zone. Management for plantations includes thinning, extended rotation cycles and clear‐cutting. For secondary forests regenerating from past clearance, traditional management varies in its intensities, from clear‐cutting as coppices to small‐scale understory clearance. Here we provide a first synthesis of published research on biodiversity in planted and secondary forests of Japan, relevant to management of these types of forest in northern temperate regions. Systematic review and meta‐analyses of papers published in English and Japanese quantified management impacts on species richness and abundance of several taxa, in relation to moderator variables including stand age and management intensity. Plantation thinning substantially increases the richness and abundance of several taxa. Effect sizes decline with time since thinning for the abundance of regenerating saplings and seedlings, necessitating repeated thinning treatments every 6 years to sustain this positive effect. Taxonomic groups exhibit variable relationships with stand age in both planted and secondary forests, indicating a need to include both young and old forest stands in managed forest mosaics. We find an insufficient evidence base is available to allow for a meaningful synthesis of low‐intensity management effects in historically managed secondary forests, with studies varying widely in scale and reported outcomes. We outline an agenda for the research community to achieve a systematic evaluation of scale‐dependent effects of traditional forest management on biodiversity.

## 1 INTRODUCTION

Natural old‐growth forests are considered irreplaceable biodiversity resources because of their long continuity and high structural diversity (e.g., Gibson et al., 2011). Whilst their strict protection represents a conservation priority in the face of forest loss and degradation worldwide, the biodiversity potential of disturbed planted and secondary forests is widely acknowledged (Putz et al., 2012). Empirical studies measuring biodiversity in forests under varying intensities of disturbance have proliferated in recent decades (Spake & Doncaster, 2017). Although several syntheses of this vast literature exist, they have limited potential for practicable recommendations across a range of contexts, because of their tendency towards narrative synthesis over quantitative meta‐analysis and a focus on charismatic taxa and tropical regions (Spake, Martin, Ezard, Newton, & Doncaster, 2015), or their inclusion of studies published only in English (Amano, González‐Varo, & Sutherland, 2016). Human‐disturbed forests particularly merit evaluation in the temperate zone, where planted and secondary forests predominate following millennia of exploitation, to the extent that only 1–2% of natural old‐growth forest remains intact in unharvested remnants (Currie & Bergen, 2008). Existing quantitative syntheses, however, typically measure the biodiversity value of disturbed forests by their comparison to natural, old‐growth forests as a reference. This conventional setup yields inferences of limited practicable value to managers and conservationists in temperate nations where little old‐growth remains, and has resulted in the exclusion of much research from pan‐global syntheses (Spake & Doncaster, 2017). Consequently, the efficacy of forest management practices for biodiversity conservation remains poorly understood for much of the temperate zone (Lindenmayer, Messier, Paquette, & Hobbs, 2015).

The Strategic Plan for Biodiversity 2011–2020 and Aichi Biodiversity Targets, adopted by the Convention on Biological Diversity in 2010 explicitly advocate implementing conservation measures within planted and secondary forests. Planted forest area is increasing globally, having risen from 4 to 7% of total forest area between 1990 and 2015. The largest increases have occurred in the temperate zone, and regionally in East Asia, Europe, and North America, for diverse purposes including production, soil protection, and carbon sequestration (Payn et al., 2015). Plantations generally lack the continuity and structural attributes typical of old‐growth forests, giving rise to the moniker of “green deserts” (Koh & Gardner, 2010); however, their habitat quality can vary in relation to management activities (Humphrey, 2005). Aichi Target 7 advocates their sustainable management in support of biodiversity. Principal management interventions for enhancing biodiversity in temperate plantations include disturbance by stand thinning to enhance the natural regeneration of native trees, and rotation age extension, on the premise that many species are dependent on later successional stages (Spake et al., 2015).

Aichi Target 15 calls for the restoration at least 15% of degraded areas through conservation and restoration activities. Secondary forests arise from both assisted restoration, and unassisted forest regeneration following human disturbance or land abandonment (Chazdon, 2008). Several options are available for their management, with traditional management receiving mounting interest because of potential benefits for biodiversity, biomass, and climate change mitigation (Müllerová, Hédl, & Szabó, 2015). Throughout the northern temperate zone, broadleaved forests that border human settlements have been managed over millennia for fuelwood, fertilizer and food (Takeuchi, 2010). Stands of trees were cut in rotations of 15–30 years, while less intensive practices included litter removal, understory clearance and tree thinning to provide fertilizer, fuel, livestock feed and bedding (Kirby & Watkins, 1998). Worldwide, traditional secondary forest management largely ceased during the mid‐1900s, as fossil fuels and chemical fertilizers became widely available (Kirby & Watkins, 1998). Abandonment of traditional management is widely regarded as a driver of biodiversity loss, because of reduced habitat suitability for early successional species requiring open habitats or vegetation structures that are reduced under heavy shade (Takeuchi, 2010). Interest is mounting in a return to traditional management at varying intensities across the temperate zone for “abandoned” public, private and even protected forests. For example, the global Satoyama Initiative (http://satoyama-initiative.org), launched concurrently with the Strategic Plan for Biodiversity in 2010, advocates the value of traditional management not only in Japan but globally. Moreover, understory clearance is traditionally practiced in urban woodlands in Europe and Japan, in compliance with recreational and aesthetic values (Heyman, 2010).

The nation of Japan provides a valuable opportunity to assess the impacts of planted and secondary forest management on biodiversity in the northern temperate zone (Supporting Information Appendix A). Forest covers approximately 25 million hectares in Japan, constituting two thirds of the total land area (Forestry Agency, 2009), but very little is pristine (Yamaura, Oka, Taki, Ozaki, & Tanaka, 2012). Plantations occupy more than 40% of total forest area, principally as monocultures of Japanese cedar (*Cryptomeria japonica*), Hinoki cypress (*Chamaecyparis obtusa*), and larch (*Larix kaempferi*), with the rest comprising secondary forests dominated by evergreen and deciduous oaks (*Quercus* sp.), and red pine (*Pinus densiflora*), naturally regenerating from past coppicing and selective cutting at varying intensities (Forestry Agency, 2009). Japan's vast forest area and interest in conservation has generated copious empirical research on biodiversity responses to forest management interventions (Higuchi & Primack, 2009). The northern temperate forestry community stands to benefit from a synthesis of this rich literature, because of the wide practice and history throughout the temperate zone of management interventions exemplified by Japanese forest management. For example, just as for Japan, the majority of Central European forests are restricted to mountain areas, and have been exploited by clear‐cutting and coppicing for millennia (Hilmers et al., 2018; Takeuchi et al., 2003). Interest in the revival of active management within “abandoned” secondary forests is increasing in Japan, as also in the United Kingdom and Europe (Takeuchi et al., 2003), while clear‐cutting of plantations of the sort that predominates in North America (Lõhmus, Rosenvald, & Lõhmus, 2006) is also increasing in Japan amidst plans to increase domestic wood supply (Forestry Agency, 2017). Effective conservation strategies for temperate forests experiencing such a range of disturbance intensities require a comprehensive and quantitative understanding of how different trophic groups vary across a wide range of successional stages and management intensities (Hilmers et al., 2018). With its forests at various successional stages following a diversity of management practices, Japan's empirical literature offers the possibility of such understanding. Many of Japan's forestry studies, however, are published only in Japanese (Nagaike, 2012), reflecting the major barrier that language still presents to the global compilation and application of scientific knowledge (Amano et al., 2016). Indeed, given the extent of its forest area and richness of empirical research, it is apparent that Japanese studies are underrepresented in synthetic studies of forest management impacts. Although several narrative syntheses exist (Inoue, 2005; Nagaike, 2012; Yamaura et al., 2012), a robust systematic and quantitative synthesis is lacking.

Here we synthesize and quantitatively assess the effects on biodiversity of coniferous and broadleaved forest management practices in Japan which are widely promoted to conserve biodiversity across the northern temperate zone. Specifically, we examine impacts on the species richness and abundance of a range of taxonomic groups from four management interventions: (a) plantation thinning; (b) extended rotation cycles (plantation age); (c) traditional management of secondary forests (secondary forest age); and (d) lower intensity traditional management of natural and abandoned secondary forests (thinning, understory clearance and/or litter removal). By synthesizing the Japanese forestry literature published in English and Japanese, we aim to provide practicable recommendations to inform policy‐making in Japan, which will be relevant also to managed forests across the northern temperate zone, because of the wide practice of the management interventions we synthesize.

## 2 MATERIALS AND METHODS

### 2.1 Literature search and data extraction

We followed standard systematic review methods (Pullin & Stewart, 2006) to collate published empirical studies on the focal management interventions. Following recommendations of Amano et al. (2016), we conducted extensive literature searches in both English and Japanese, using Web of Science, Google Scholar, and J‐Stage (the largest platform for publishing electronic journals in Japan; https://www.jstage.jst.go.jp/). We sought studies conducted in Japan using search terms relating to the country, forest management interventions, and biodiversity (search queries in Supporting Information Appendix B). Additional literature was identified by “snowballing”: searching for references within retrieved articles and reviews. We used the R package “metagear” (Lajeunesse, 2016), to screen retrieved abstracts.

Relevant studies compared biodiversity in planted or secondary forest with appropriate controls, given in Table 1. In order to produce data relevant to the management decisions affecting forest management actions in Japan and the rest of the temperate zone, we compared the biodiversity value of forests in their current state (control groups of low disturbance relative to treatment groups), compared to a plausible alternative state (treatment groups of managed forests; Table 1; Spake & Doncaster, 2017). Species richness was used as a proxy for biodiversity, being most widely used biodiversity measure (Magurran, 2004). We note here that authors measuring “species richness” in primary studies were actually measuring species density, the number of species per unit area (Gotelli & Colwell, 2001), wherein richness is standardized against area or sampling effort across treatments. We use the term species richness to avoid confusion with abundance, which is often measured as density (number of individuals per unit area). We evaluate the implications of this diversity metric in our discussion.

**Table 1:** Descriptors of treatment and control groups used in our systematic review of the impact of four forest management interventions on biodiversity in Japan

Management intervention	Treatment group	Control group	Moderator variables considered in quantitative synthesis
***Plantation forest***			
Thinning	Thinned plantation.	Unthinned plantation.	Taxon, stand age, taxon, thinning intensity, canopy dominant, thinning intensity.
Extended rotation cycles (stand age).	Young plantation (1–50 years, median age 21 years).	Overmature plantation beyond economic felling age (typically >65 years).	Taxon, treatment stand age, canopy dominant.
***Secondary forests***			
High‐intensity traditional management of secondary forests (stand age).	Secondary forests regenerating from stand‐level clearance such as through coppicing.	Unmanaged secondary forest that has not been cleared for >100 years.	Taxon, treatment stand age, canopy dominant.
Lower intensity traditional management of natural and secondary forests.	Natural or secondary forest that has undergone recent thinning, understory removal and/or litter removal.	Recently unmanaged or “abandoned” secondary or natural forest.	—

## 3 META‐ANALYSIS OF PLANTATION THINNING EFFECTS ON BIODIVERSITY

To ensure meaningful comparisons across studies, we sought publications that compared thinned treatment to unthinned control stands with the same canopy dominant and age. For each comparison of species richness and/or abundance, the effect size of log response ratio (ln*R*) was calculated, as:

(1) $$\ln R\;=\;\ln\left({\bar{x}}_{T}\right)\;-\;\ln\left({\bar{x}}_{C}\right),$$

where ${\bar{x}}_{T}$ is the mean species richness or abundance in treatment forest stands and ${\bar{x}}_{C}$ is the mean value for control stands. The ln*R* describes the proportional difference in species richness or abundance between control and treatment groups. The natural log transformation of the response ratio linearizes the metric, treating deviations in the denominator and the numerator as equal, and normalizes its otherwise skewed distribution (Hedges, Gurevitch, & Curtis, 1999). Abundance measures included values of cover, biomass, and number of individuals. If an article reported single abundance values for a number of different species within the same taxonomic group (e.g., within understory plants), we calculated the combined effect size for the group (Borenstein, Hedges, Higgins, & Rothstein, 2009).

We extracted data on the following moderator variables: taxon (understory plants, saplings and seedlings, invertebrates), thinning stand age, years since thinning and thinning intensity (percentage volume of trees removed). We used linear mixed models to investigate variation in effect size with the moderator variables. We included quadratic or log_10_ relationships with thinning intensity to test for possible nonlinear relationships. Study quality varied widely regarding replication and spatial interspersion of treatments. Meta‐analyses conventionally weight effect sizes by the inverse of study variance to account for differences in sampling effort. With forest biodiversity studies however, the variance of replicate means is often (a) unreported, (b) unavailable because sample size is one, or (c) not meaningful because studies varied widely in their design, with a high prevalence of pseudoreplicated designs (Spake & Doncaster, 2017). We accounted for differences in study quality by weighting effect sizes based on their true sample sizes, following Mayerhofer, Kernaghan, and Harper (2013) and Doncaster and Spake (2017), with the relative weights estimated as:

(2) $$\mathrm{wt}\;=\;\left(N_{C}N_{T}\right)/\left(N_{C}\;+\;N_{T}\right),$$

where *N*_*C*_ and *N*_*T*_ are the true sample sizes of the unthinned control and thinned treatments, respectively, identifying the number of spatially interspersed replicates of forest treatments (Halme et al., 2010). See Supporting Information Appendix D for this weighting rationale.

Where articles reported separate values for two or more study locations, canopy dominants, or taxonomic groups, we regarded each as an independent observation. Study was included as a random effect to accommodate studies with multiple effect‐size estimates based on a common control stand. Just one study was omitted from the abundance analysis, because of a zero abundance that precluded calculation of a response ratio. All possible additive models were constructed by maximum likelihood methods using packages lme4 (Bates, Maechler, Bolker, & Walker, 2014), and MuMIn (Barton, 2013). Akaike's Information Criterion (AIC) with small sample correction (AICc) was used to identify a candidate set (Burnham & Anderson, 2004). We performed model averaging when multiple models were plausible (ΔAICc <4), but selected a single model if the next best model had ΔAICc >4. Goodness of model fits was estimated by the marginal *R* ^2^ (Nakagawa & Schielzeth, 2013).

### 3.1 Meta‐analysis of extended rotation cycles for plantations and high‐intensity traditional management of secondary forest

Studies investigating the impact of extended rotation cycles typically measure biodiversity in plantations of varying ages (Supporting Information Appendix D). Studies investigating the impact of high‐intensity traditional management typically measure secondary stands of varying ages following clearance (Supporting Information Appendix E). We here define secondary forests sensu *lato* as any forests regenerating naturally from a stand‐level clearance event, following high‐intensity coppicing or clear‐cutting. For plantations, we included studies that used a control group of overmature plantations beyond 50 years of age (median reference stand age: 76 years), reflecting the Japanese convention to harvest at approximately 50 years old (Forestry Agency, 2017). For traditional secondary forests, we included studies that used at least one reference stand exceeding 100 years in age (median age: 128 years).

Whilst all studies of stand age‐biodiversity relationships included a common reference stand, studies varied in treating stand age as either a continuous or categorical variable. When treated as continuous, researchers sampled forest plots across a stand‐age gradient, whereas when treated as categorical, researchers sampled plots replicated within grouped age classes. We therefore adopted the meta‐analytical approach of previous stand‐age biodiversity syntheses by Curran, Hellweg, and Beck (2014) and Martin, Newton, and Bullock (2013), which synthesized pair‐wise comparisons between single treatment stands and either replicated or unreplicated reference stands. We calculated ln*R* as the effect size for abundance and richness comparisons. We grouped taxa into the following groups: butterflies and moths, bees and wasps, terricolous invertebrates (beetles, spiders, and ants sampled above the soil), soil invertebrates (collembola and mites sampled from within soil), fungi and ground‐layer plants (including groups termed as vascular, shrub, or herb species).

We used linear mixed models to investigate variation in effect size (ln*R*) with stand age and other moderator variables. Just one study was omitted from the abundance analysis, because of a zero abundance. Moderator variables included taxon, stand age, and their interaction. We included quadratic or log_10_ relationships with stand age to test for possible nonlinear biodiversity recovery, and we weighted by sample size as described above. Study was included as a random effect to accommodate publications with multiple effect‐size estimates using a common control. To identify important moderators of effect size differences across studies, we used model selection as described above. All quantitative analyses were performed using R (R Core Team, 2018).

### 3.2 Review of lower intensity traditional management impacts on biodiversity in natural and secondary forests

We compiled studies that assessed the influence on biodiversity in secondary forests of traditional management practices that are lower in intensity than clear‐cutting in terms of the amount of biomass removed (Supporting Information Appendix F). Such practices include tree thinning, understory clearance and litter removal (Shibuya, Kubota, Kikvidze, & Ohsawa, 2008). We selected studies that compared forest sites described as recently unmanaged, abandoned or natural to forest sites that were either currently or recently actively managed (within 20 years). To control for stand age and canopy‐dominant effects on biodiversity, we sought studies with similar control and treatment stands in these attributes. Each study was described according to the following moderator variables: taxonomic group, stand age, years since management, and management type (thinning/understory removal/litter removal) and intensity (e.g., volume of trees removed, area of understory cleared). Descriptions of management actions were often vague, with inconsistent reporting of intensity and stand age. Insufficient replication and crossing at the level of management intervention by taxonomic group precluded meaningful quantitative summaries. We therefore synthesized these results narratively.

## 4 RESULTS

### 4.1 Effects of plantation thinning on richness and abundance

Twenty‐six publications were retrieved (Supporting Information Appendix C) comparing biodiversity in thinned and unthinned plantations, spanning a range of thinning intensities and taxonomic groups on widely distributed studies across Japan. Plantations in all but one study were planted as monocultures. One study was retrieved on birds (Toyoshima, Yamaura, Yabuhara, & Nakamura, 2013), and one on below‐ground mite communities (Takasaki, Takenaka, & Yoshida, 2010). Studies on insects comprised Hymenoptera, Coleoptera, Diptera, and Lepidoptera. Data were sufficient for meta‐analysis of saplings and seedlings, ground‐layer plants and insects (pooling across Orders), totaling 65 richness and 134 abundance comparisons.

**Figure 1:**
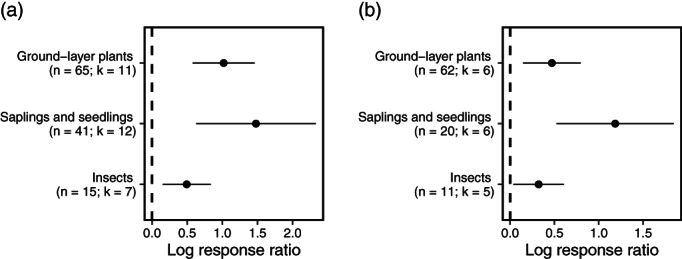
Summary mean effects and confidence intervals of plantation thinning on a) abundance and b) species richness of biotic communities, from n studies at k study sites

**Figure 2:**
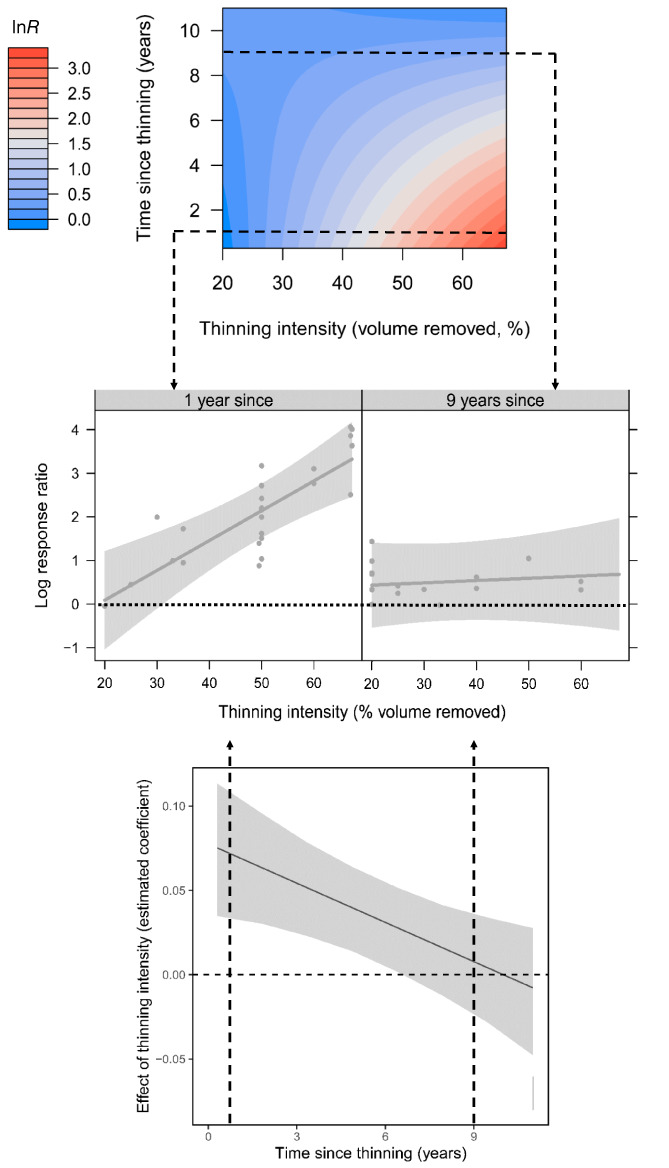
Impacts of plantation thinning on abundance of saplings and seedlings as dependent on time since thinning. a) Countour plot showing abundance differences (lnR) between thinned and unthinned forest stands as a function of thinning intensity and time since thinning. Dashed arrows point to sections through the plot at 1 and 9 years, illustrated in part b below. b) Influence of thinning intensity on abundance differences at 1 and 9 years since thinning, showing grey‐shaded 95% CI in the regression based on between‐study uncertainty in fixed effects only; values above horizontal dotted line signify higher abundance in thinned than unthinned stands. c) Marginal effect of thinning intensity, conditional on years since thinning; shading as for part b. Dashed arrows at 1 and 9 years show effects corresponding to response ratios in part b above. No effect of thinning intensity is detectable after six years. Regression used coefficients of the minimum adequate model

**Figure 3:**
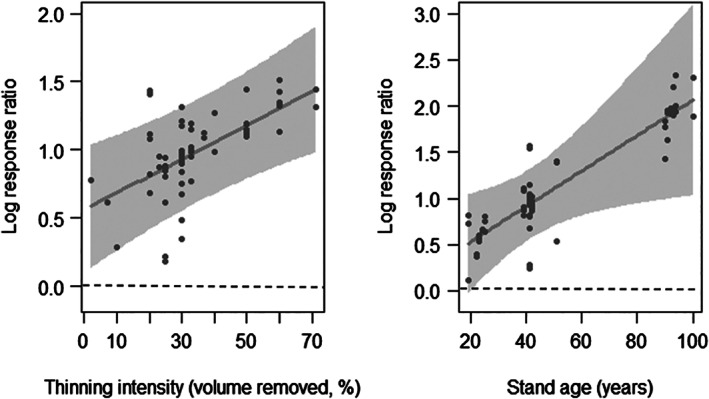
Influence of a) thinning intensity and b) stand age on abundance differences between thinned and unthinned plantation stands for understorey plants (horizontal dashed line means no difference). Regression used coefficients of the minimum adequate model. Grey shading shows 95% prediction intervals based on between‐study uncertainty in fixed effects only

Subgroup analysis showed that plantation thinning substantially increased the richness of tree saplings and seedlings, ground‐layer plants and insects by 227, 60, and 38% respectively (*p* < 0.05; Figure 1(a)). Thinning also increased abundance of the studied groups, by 330%, 177% and 63% respectively (*p* < 0.05; Figure 1(b)). Effect sizes were heterogeneous amongst studies, because of study‐wise variation in stand ages and thinning intensities. Collinearity between taxonomic group, stand age and thinning intensity restricted our mixed effects modeling of abundance effect sizes to broadleaved saplings and seedlings and ground‐layer plants only. Moderator collinearity precluded mixed‐effects analysis of species richness effect sizes. The most important predictors of sapling and seedling abundance were thinning intensity, time since thinning and their interaction (Supporting Information Appendix C, marginal *R* ^2^ = 0.42 for minimum adequate model). Impacts of thinning on abundance depended on both the volume of trees removed and time since thinning (Figure 2(a)). In recently thinned stands, the impacts increased linearly with increasing volume of trees removed. This effect declined with the time elapsed since thinning (Figure 2(b)), with no effect detectable after ~6 years (Figure 2(c); Supporting Information Appendix C). Effect sizes of ground‐layer plant abundance increased with both thinning intensity and plantation stand age, with a thinning intensity of about 66% resulting in a doubling of ground‐layer plant abundance (Figure 3; Supporting Information Appendix C, marginal *R* ^2^ = 0.23 for minimum adequate model).

### 4.2 Effects of plantation age on species richness and abundance

**Figure 4:**
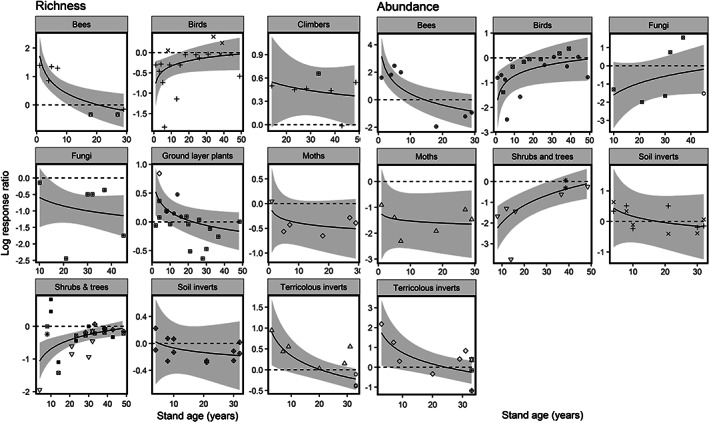
Influence of stand age on species richness and abundance effect sizes in planted stands relative to extended rotation planted stands. The horizontal dashed lines reference zero difference between extended rotation and younger treatment forest stands. Regressions used coefficients of the minimum adequate model. Grey shading shows 95% prediction intervals based on between‐study uncertainty in fixed effects only. Different symbols correspond to different publications

**Figure 5:**
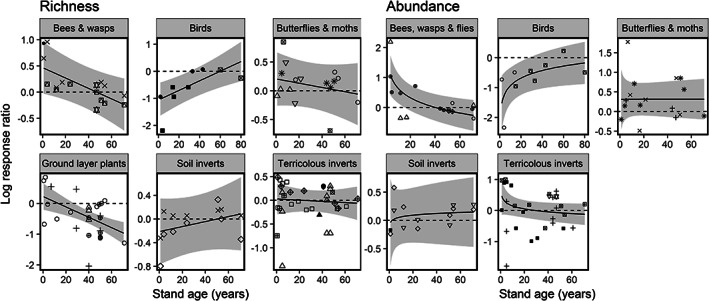
Influence of stand age on species richness effect sizes in younger secondary forest stands relative to older (>100‐yr) abandoned forest stands. The horizontal dashed lines reference zero difference between extended rotation and younger treatment forest stands. Regressions used coefficients of the minimum adequate model. Grey shading shows 95% prediction intervals based on between‐study uncertainty in fixed effects only. Different symbols correspond to different publications

We retrieved 15 publications (Supporting Information Appendix D) describing species richness or abundance differences between younger planted and reference overmature planted forest stands, yielding 115 richness and 68 abundance comparisons. One study was excluded from the quantitative synthesis because of its outlying age of 20–250‐year‐old stands (Suzuki, Suzaki, Okumura, & Ikeda, 2005). The minimum adequate models selected to explain differences in species richness and abundance between extended rotation and younger planted forest stands included taxonomic group (species richness: *F*_1,85_ = 5.99; *p* < 0.001; abundance: *F*_1,66_ = 11.63; *p* < 0.001), log_10_ stand age (species richness: *F*_1,88_ = 4.11; *p* = 0.046; abundance: *F*_1,66_ = 0.02; *p* = 0.875), and their interaction (specie richness: *F*_1,90_ = 3.85; *p* < 0.001; abundance: *F*_1,66_ = 7.77; *p* < 0.001). These models had marginal *R* ^2^ values of 0.54 and 0.55 for effect sizes of species richness and abundance, respectively, and both had ΔAIC >4 to the next best model. Climbing plants, and terricolous invertebrates exhibited consistently higher species richness and abundance levels in younger planted stands than overmature planted stands, while fungi were consistently richer and more abundant in overmature stands (Figure 4). The interaction reflects taxon‐specific relationships of species richness and abundance with stand age (explaining the absence of a detectable stand‐age main effect). Birds, shrubs, and trees demonstrated positive relationships, whilst flying and terricolous invertebrate and ground layer plant richness had declining abundance with stand age (Figure 5). Fungi richness and abundance did not vary detectably with stand age (Figure 4).

### 4.3 Effects of traditional management of secondary forests on richness and abundance

We retrieved 25 publications describing species richness or abundance in secondary forest stands regenerating from clear‐cutting, in comparison to a reference secondary forest stand >100 years old (Supporting Information Appendix E), yielding 141 richness and 105 abundance comparisons. Prior to modeling the relationship between richness recovery and stand age across taxonomic groups, we removed studies on trees, epiphytic plants (climbers and vines) and Diptera, because treatment forest sites from these studies did not present a stand age gradient. For the same reason, we removed studies of ground‐layer plants when modeling abundance effect sizes. The minimum adequate models selected to explain species richness and abundance differences between abandoned older and younger secondary forest contained taxonomic group (species richness: *F*_5,58_ = 5.67; *p* = <0.001; abundance: *F*_5,50_ = 6.44; *p* ≤ 0.001), stand age (species richness: *F*_1,82_ = 0.31; *p* = 0.575; abundance: *F*_1,66_ = 0.33; *p* = 0.566), and their interaction (species richness: *F*_4,82_ = 7.20; *p* = <0.001; abundance: *F*_4,66_ = 4.15; *p* = <0.01). These models had marginal *R* ^2^ values of 0.35 and 0.38 for species richness and abundance effect sizes respectively, and both had ΔAIC > 4 to the next best model. The interaction reflects a balanced opposition of stand‐age effects amongst taxonomic groups (explaining the absence of detectable main effects; Figure 5). Flying invertebrates and ground‐layer plant richness benefited from early successional conditions and declined with stand age, whilst soil invertebrates and birds exhibited lower richness levels in younger secondary forest relative to older abandoned forest and tended to increase with stand age (Figure 5).

### 4.4 Effects of lower intensity traditional management of secondary forests on biodiversity

We retrieved 27 publications that investigated the impact of low‐intensity forest management on biodiversity by comparing unmanaged and managed forest (Supporting Information Appendix F). Wide variation in study variables including management intensity, taxonomic group, and forest ages precluded a quantitative synthesis; we instead report the results narratively, in detail in Supporting Information Appendix F. Broadly, management interventions comprised tree thinning at varying intensities, understory removal and litter removal, and various combinations of these interventions. The majority of studies were observational, with researchers selecting forest sites already subjected to management by voluntary citizens or the prefectural government for purposes including compost production, mushroom production, recreation, and biodiversity conservation, with few studies performing controlled experiments (but see Shibuya et al., 2008). Studies varied widely in scale, in terms of the sizes of forest stands and the observational plots for management and biodiversity measurement (Supporting Information Table F1), and such scale information was typically not reported.

## 5 DISCUSSION

Our meta‐analysis of relatively large sets of published studies from Japan demonstrates that diverse stand‐level factors influence the impacts on biodiversity of management interventions that are widely practiced in planted and secondary forests across the northern temperate zone. Quantitative syntheses, which allow the testing of relationships between individual study outcomes and their characteristics, have demonstrated variable and nonlinear responses amongst taxa to important management covariates (time since thinning, thinning intensity, stand age). These are not typically treated by other meta‐analyses, which tend simply to compare “managed” and “unmanaged” forests (e.g., Chaudhary, Burivalova, Koh, & Hellweg, 2016; Paillet et al., 2010; Verschuyl, Riffell, Miller, & Wigley, 2011). Broadly, our results demonstrate that both young and old stands must be present in managed forest mosaics to support multiple taxonomic groups. Our comprehensive review has revealed the need for a stronger evidence base to evaluate the impacts on biodiversity of traditional low‐intensity management strategies for northern secondary temperate forests.

### 5.1 Effects of plantation management on biodiversity

Under the Forest and Forestry Revitalisation Plan to increase Japan's timber self‐sufficiency (Nagasaka, Bocher, & Krott, 2016), mature plantations are increasingly subject to clear‐cutting in many parts of Japan including Kyushu and eastern Hokkaido. In topographically complex areas that preclude commercial management, a range of groups under municipal control, including voluntary NGOs and private companies, funded by a national Forest Environment Tax to be introduced this year (Forestry Agency, 2017), conducts plantation thinning. The aim of such management is to enhance ecosystem services that benefit the public, including watershed conservation, mitigation of climate change in addition to biodiversity conservation, and the assisted regeneration of natural broadleaved forest (Forestry Agency, 2017). Our results of plantation forest age and thinning impacts on biodiversity can provide some guidance to practitioners conducting such management.

Plantation thinning positively affected species richness and abundance of invertebrates, ground layer plants and saplings and seedlings (Figure 1), which is consistent with a synthesis of thinning impacts on several taxa, including birds mammals and invertebrates, in North American forests (Verschuyl et al., 2011). Thinning substantially increased sapling and seedling abundance and richness. The response of sapling and seedling abundance to thinning intensity depended on the time since thinning; effect sizes increased with thinning intensity in recently thinned plantations, but no effect of intensity was detectable in stands thinned approximately 6 years previously (Figure 2; Supporting Information Appendix C). This finding suggests repeated thinning at such intervals is required to ensure the survival of regenerated seedlings. The effect of thinning on understory plant abundance increased linearly with stand age (Figure 3(b)), demonstrating a larger effect in older stands with more closed canopies, suggesting such stands could be prioritized for thinning efforts. Effect size relationships with thinning intensity and stand age for saplings/seedlings and understory plants did not plateau, suggesting an absence of generally applicable thresholds for maximizing positive effects on species richness or abundance.

Our explanatory power was low for models explaining understory abundance with thinning intensity and stand age, and previous research has shown that thinning resulted in almost no increase in understory vegetation. Such instances are attributed to grazing by deer (Tamura & Yamane, 2017), and so the effectiveness of thinning in Japan and other northern temperate nations that similarly are experiencing high deer densities (Côté, Rooney, Tremblay, Dussault, Waller, 2004), will likely depend on local deer densities and grazing management.

Timber harvesting in Japan has conventionally used a rotation age of between 40 and 60 years (Masaki et al., 2006), similar to other northern temperate nations (Benkman, 1993; Macdonald, Gardiner, & Mason, 2010). However, extended rotation ages >70 years are becoming increasingly widespread, to hedge against fluctuations in timber prices with a larger, more valuable stock (Masaki et al., 2006). This review has shown that the richness and abundance of birds, shrubs and trees increases nonlinearly with planted stand age (Figure 5), likely attributable to the combined effects of time favoring colonization by dispersal‐limited species, and structural diversification as forests age (Norden & Appelqvist, 2001). The richness of several taxonomic groups, including ground‐layer plants and soil and terricolous invertebrates, declined with stand age, however. With different taxonomic groups exhibiting contrasting species richness levels in different successional stages, our results from Japan support recommendations for both young and old forest stands to be included in plantation forest mosaics for invertebrate and ground‐layer plant conservation (Viljur & Teder, 2016).

We note that low sample sizes for some taxonomic groups led to low precision in effect sizes for these groups, including the climbing plants, moths, and fungi. Although functional groups within broad taxonomic groupings vary in their responses to forest management impacts (Spake et al., 2015), low sample sizes forced their grouping into broad taxonomic classifications, which may have obscured differences in responses of finer classifications. Richness and abundance relationships with stand age revealed little difference between the biodiversity value of 50‐ and approximately 70‐year‐old (control) plantations. Extending the conventional rotation age to >70 years may therefore not offer a win‐win for economic return and biodiversity conservation. However, it is important to understand patterns of biodiversity variation beyond 70 years, because a large quantity of planted stands is likely to remain unharvested in Japan. We retrieved one study of hinoki cypress plantations from 20 to 250 years old. Suzuki et al. (2005) found that stands >200 years old differed in species compositions to stands <100 years old, having developed multi‐layered canopies with broadleaved species occupying the lower canopy. The authors state that canopy openings were a product of both past logging and natural stand dynamics, and suggest that much older planted forests stands may require thinning.

### 5.2 Effect of traditional secondary forest management on biodiversity

The Satoyama Initiative promotes the revival of traditional management at a range of intensities, based on presumed benefits to biodiversity and ecosystem services (Takeuchi, 2010). This includes the return of more traditional interventions, with secondary forests managed by clear‐cutting, in addition to more commonly practiced contemporary interventions that include thinning, undergrowth clearance and leaf‐litter removal applied at smaller extents (Shibuya et al., 2008). Contemporary satoyama management practices reflect shifting sociocultural and resource needs. Although the original primary function of satoyama was for production, interest is mounting in the value of satoyama for delivering a range of other ecosystem services. In particular, the cultural and aesthetic opportunities inherent in the woodland management process are motivating community‐based management, with subsidies from municipal governments (Tatsui & Fujii, 2006; Yokohari & Bolthouse, 2011). Here we set the results of our analyses of secondary‐forest management in the context of future research needs that are required to effectively guide local community groups in maintaining secondary forest biodiversity.

We have shown that species richness and abundance relationships with stand age (a proxy for high‐intensity traditional management) varied according to taxonomic group in secondary forests. Flying invertebrates and ground‐layer plants benefited from more open, early‐successional conditions and tended to decline with stand age while the abundance and richness of birds and soil invertebrates increased with stand age. Late successional secondary forests are needed to support mature forest‐specialists, but are scarce in southern Japan and much of Europe because of the long history of forest usage (Currie & Bergen, 2008; Totman, 1989). Our results accord with the taxon‐specific responses to stand age found in Europe and North America (Spake et al., 2015), and also suggest that managed mosaics should contain secondary forests that remain set‐aside to mature.

The studies retrieved by our review of low‐intensity traditional management varied widely in taxonomic group (plants, invertebrates, and fungi), the particular combination of management interventions and their intensity (amount of biomass removed), scale of management and study design; with positive, negative, and neutral effects of management on a range of biodiversity metrics reported (Supporting Information Table S1). Because of this variation, it is difficult to make clear generalizations on the effect of low‐intensity traditional management on forest biodiversity. It can only be concluded that impacts are highly heterogeneous and context‐specific, with positive, negative, or neutral management effects on a range of biodiversity metrics. We therefore caution against the promotion by governments of the biodiversity value in traditional forest‐management practices, such as through the Satoyama Initiative, until an understanding of the effects of scale‐dependent management interventions is achieved (Spake et al., 2019). We note, however, that management can provide other benefits including the enhancement of cultural ecosystem services.

### 5.3 Ensuring the maintenance of forest biodiversity within Japan's managed forests: A research agenda

Effect sizes were estimated from species density, as opposed to species richness, which is typically estimated by using abundance or incidence distributions to model the number of undetected species (Gotelli & Colwell, 2001). It is therefore possible that species density differences could have been partly driven by effects on overall abundance of individuals. Additionally, using species density can potentially underestimate the true biodiversity difference between, for example, >100‐year‐old secondary and younger secondary forests; higher intrinsic richness in older forests could lead to a systematic under‐sampling bias that misses more species per site than in younger secondary sites when sampling is standardized by area or sampling effort (Spake & Doncaster, 2017). Moreover, use of species density or richness alone does not account for compositional differences between forest stands, and ignores the incidence of rare or functionally important species, and important attributes such as invasiveness. Much research into biodiversity–ecosystem functioning relationships has shown that biodiversity (including taxonomic, functional, and phylogenetic diversity) affects the functioning of ecosystems (e.g., primary production, decomposition, nutrient cycling, trophic interactions, and so on) and consequently a range of ecosystem services (e.g., food production, climate regulation, pest control, pollination etc; Cardinale et al., 2012). An important next step is to use biodiversity data from forests to give practical advice for enhancing ecosystem functioning and ecosystem services (Mori, Lertzman, & Gustafsson, 2017).

The studies included in our meta‐analysis varied widely in scale, in terms of the size of the individual sampling plots (grain), the area of inference represented by each data point (e.g., whether a forest stand), the scale at which a mean is calculated (the focus), and the size of the study area (extent; Gerstner et al., 2017). While grain was frequently reported, focus and extent were often missing, or descriptions did not allow clear distinctions among the spatial scale components, precluding an analysis of the scale‐dependence of effect sizes. We follow Gerstner et al. (2017) in urging studies to report such information in the future to allow for an understanding of the importance of management and study scale of the effects of forest management on biodiversity. We see a need for researchers to conduct more systematic field studies of the impacts of different traditional management practices on biodiversity across Japan. This can be achieved by evaluations of practices piecemeal (thinning, understory clearance, and litter removal), and in combination across a network of well‐replicated sites across Japan. There is a particular need to consider and report the extent of the area managed, and the effect of the intensity of management practices (e.g., volume of trees removed), so that thresholds may be identified to guide management practices.

It is conceivable that climate could moderate forest management impacts on biodiversity. For example, the importance of plantation thinning for increasing sapling abundance could depend on variables such as temperature and solar radiation, with a certain level of canopy cover required to buffer against climatic extremes. Testing for both additive and interactive effects of climatic variables on effect sizes (management impacts) would require a larger sample size than is currently available, and represents a future research opportunity for Japan.

In conclusion, we call for a coordinated research agenda to achieve a systematic evaluation of traditional management impacts, at a range of intensities and scales, on forest biodiversity and ecosystem functioning in Japan. Future studies of all forests should measure community attributes other than species richness that capture ecosystem function, in addition to reporting attributes of scale and the topographic and regional climatic context, to permit testing for interacting effects. Japan presents an ideal opportunity to test for such ‘cross‐scale interactions’ (Peters, Bestelmeyer, & Turner, 2007), because of its wide climatic and topographic gradients that could potentially modify biodiversity responses to the management of its vast forest estate (Spake et al., 2019; Yamaura, Amano, Kusumoto, Nagata, & Okabe, 2011).

## CONFLICTS OF INTEREST

The authors declare no potential conflict of interests.

## AUTHOR CONTRIBUTIONS

RS, CPD, and KKi conceived the idea. RS, CPD, YY, and KKi designed the analytical approach. RS, SY, and KKa collated the data. RS carried out all statistical analyses. RS, CPD, and YY wrote the manuscript. All authors discussed the results and contributed to the manuscript.

## Supporting information

Supporting Information
