# Supplementary material for: Meta‐analysis of management effects on biodiversity in plantation and secondary forests of Japan
Source: Conserv Sci Pract. 2019 Mar 20;1(3):e14. doi: 10.1111/csp2.14 (PMC8611798; doi:10.1111/csp2.14)
Supplement: Supplementary file 1 — Supporting Information [file CSP2-1-e14-s001.docx]

# Supporting information

## Appendix A – Elaboration of the history and ecology of forest management in Japan

### Forest extent and management in Japan

The majority of Japan’s forest area has been managed in the past, and is now dominated by coniferous plantations and secondary broadleaved forests. Planted forests constitute over 40% of forest area (FRA, 2010), with the majority established during 1950-1980, by replacing grasslands and natural broad-leaved forests with high-productivity conifers, namely Japanese cedar (*Cryptomeria japonica*), Hinoki cypress (*Chamaecyparis obtusa*), and larch (*Larix kaempferi*) to meet increased housing and timber demands following World War II (Yamaura et al., 2012). Secondary forests comprise more than 50% of Japan’s forest area. Much of the ancestral forest is believed to have been heavily exploited during Japan’s pre-historic era, with deforestation continuing into the second half of the last millennium for agricultural developments and collecting building materials and fuelwood for manufacturing iron. Secondary broad-leaved forests near to villages were later clear-cut as coppices over cycles of 15 to 30 years, to provide a source of raw materials including charcoals, and the undergrowth was cleared for fuel, compost and edible mushroom production up until a few decades ago (Takeuchi, 2010, Yokohari and Bolthouse, 2011; Chen & Qiu, 2012). These young secondary broad-leaved forests are now called ‘satoyama’ (literally meaning hill forests near villages), and they epitomise the cultural landscape in warm to cool-temperate Japan and Asia Pacific regions (Kwon et al. 2006).

The vast majority of both planted and secondary forests in Japan have remained unmanaged since the 1970s, for socioeconomic reasons. These include the high cost of harvesting plantations due to topographic complexity and uncontrolled bamboo growth, the importation of low-cost timber from North America and South East Asia, and difficulties with co-ordinating the management of small-scale privately owned forests, which make up 58% of forest area (Yamaura et al., 2012; Forestry Agency, 2014). The cessation of satoyama management across Japan coincided with large-scale urban development and the ‘fuel revolution’, in which rapid economic development led to the replacement of fuelwood and green fertiliser with imported fossil fuels and chemical fertilisers (Iwata et al., 2011).

### Management interventions proposed for Japan

The future management of Japan’s forests is currently being discussed by the forestry community in Japan (e.g. Forest Agency, 2016; Yamaura et al. 2012). Japan’s NBSAP 2012-2020 considers the underuse of the nation’s forest resources as responsible for a biodiversity ‘crisis’ (JBO, 2016). Broadly, it proposes that revived exploitation of planted and secondary forests will benefit biodiversity by promoting species dependent on early successional habitats.

#### Plantation forests

a high proportion of planted forests are now at economic felling age, between 40 and 60 years of age (Yamaura et al., 2012), and several options are being discussed for their management (Forestry Agency, 2016). Under the Forest and Forestry Revitalisation Plan to increase Japan’s timber self-sufficiency rate (Nagasaka et al. 2016); mature plantations are now actively cut in many parts of Japan including Kyushu and eastern Hokkaido. On the contrary, Japan’s NBSAP 2012-2020 proposes the reversion of planted forests back to natural forests (Ministry of Environment, 2012). Plantation thinning, the dominant silvicultural activity in recent decades under the “Utsukushii Mori Zukuri” national campaign (National Movement for Fostering Beautiful Forests), has been proposed to enhance the natural regeneration of broadleaved tree species by improving light conditions and biodiversity of various animal groups, as well as rotation age extension for fostering more economically valuable, larger logs (Ministry of Environment, 2012; Yamaura et al. 2012).

#### Secondary forests

For secondary broadleaved forests, the collapse of the satoyama regime in Japan triggered a reassessment of its value during the last decade (Takeuchi, 2010). Japan’s National Biodiversity Strategy and Action Plan 2012-2020 (NBSAP) advocates the promotion of national and international initiatives that have been launched to restore satoyama, not only in Japan, but throughout the Asia-Pacific region, due to perceived benefits for biodiversity conservation (such as the International Partnership for the Satoyama Initiative; <http://satoyama-initiative.org/>). This includes the return of more traditional interventions, with secondary forests managed by clear-cutting, in addition to more commonly practised contemporary interventions that include thinning, undergrowth clearance and leaf-litter removal applied at smaller extents (Shibuya et al. 2008). Such management by local governments is limited outside of national parks due to insufficient budgets and ecological knowledge, and local voluntary community groups play a prominent role (Tsuchiya et al., 2013, 2014). Contemporary satoyama management practices reflect shifting sociocultural and resource needs; whilst the previous primary function of satoyama was for production, interest is mounting in the value of satoyama for delivering a range of other ecosystem services. In particular, the cultural and aesthetic opportunities inherent in the woodland management process are motivating community-based management (Yokohari & Bolthouse, 2011; Tatsui & Fujii, 2007). Indeed, a common idiom spoken of forests in Japan, “森林は手入れをしないと山が荒れる” translates literally to “the mountain is rough if not cared for”, and culturally implies that forests are degraded without management, with only managed forests maintaining their aesthetic appeal. However, whether preventing succession with satoyama management can actually enhance biodiversity has not been investigated systematically.

In addition to these stand-scale management propositions for planted and secondary forests, landscape-scale options are also under consideration. For example, Japan’s NBSAP 2012-2020 recognises that restoring well-connected ecological networks is essential for implementing biodiversity conservation in urbanized lowlands of Japan, where fragmented forest remnants in the precincts of shrines and temples are scattered among residential areas, rice paddies and farmland.

## **Appendix** B – Keywords used for search in ISI Web of Knowledge

Relevant studies were identified through computerised searches in the ISI Web of Science database (all databases). An exhaustive search for Japanese papers using words related to biodiversity and forests was done in J-Stage and Google Scholar. The finalised search query for the ISI Web of Knowledge performed on 01 July 2017 was:

CU= (Japan)

AND

TS= (biodiversity OR diversity OR richness OR abundance OR “species density”)

AND

TS= (forest OR coppice* OR woodland)

AND

TS=(primary OR old-growth OR oldgrowth OR “old growth” OR natural OR mature OR manag* OR clear-cut OR clearcut OR coppic* OR abandon* OR active OR passive OR restor* OR succession OR secondary OR “stand age” OR chronosequence OR plantation OR “planted forest” OR satoyama OR thinn* OR logg* OR “undergrowth removal” OR “undergrowth clearance” OR “understory removal” OR “understory clearance”)

## Appendix C: Supporting information for the review of the effects of thinning on biodiversity

#### Studies that measured biodiversity responses to plantation thinning


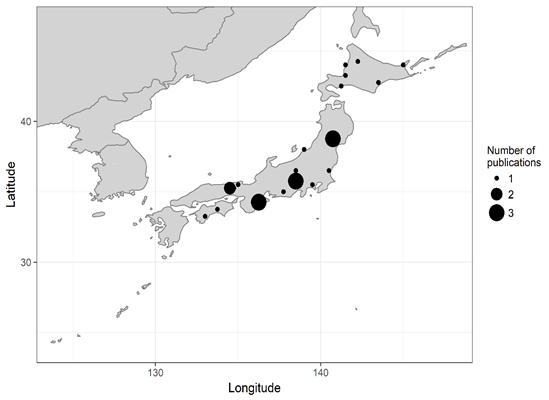


Figure C.1. Distribution of publications reporting biodiversity responses to plantation thinning retrieved by this review.

1. Ehara, H., Ishii, H., Maeto, K. 2011. Ant Community Structure and Related Environmental Factors after Line Thinning in Japanese Cedar (Cryptomeria japonica) Plantations. Journal of the Japanese Forest Society, 94(1), 36-41.
2. Hirata, A., Sakai, T., Takahashi, K., Sato, T., Tanouchi, H., Sugita, H., Tanaka, H. 2011. Effects of management, environment and landscape conditions on establishment of hardwood seedlings and saplings in central Japanese coniferous plantations. Forest Ecology and Management, 262(7), 1280-1288.
3. Ishii, H. T., Maleque, M. A., Shingo, T. G. 2008. Line thinning promotes stand growth and understory diversity in Japanese cedar (Cryptomeria japonica D. Don) plantations. Journal of Forest Research, 13(1), 73-78.
4. Kawanishi, M., Komatsu, T., Sakio, H., Yonebayashi, C. 2008. Effect of Thinning and Litter Removal on the Recovery of Riparian Vegetation in a Cryptomeria japonica Plantation. Journal of the Japanese Forest Society, 90(1), 55-60.
5. Kon, H., Watanabe, I., Yasaka, M. 2007. Effect of thinning on natural regeneration of broad-leaved trees in Abies sachalinensis plantations. Journal of the Japanese Forest Society, 89, 6, 395-400.
6. Maleque, M.A., Ishii, H.T., Maeto, K. Taniguchi, K. 2007. Line thinning enhances diversity of Coleoptera in overstocked Cryptomeria japonica plantations in central Japan. Arthropod-Plant Interactions. 1: 175. doi:10.1007/s11829-007-9016-1
7. Maleque MA, Ishii HT, Maeto K, Taniguchi S (2007) Line thinning fosters the abundance and diversity of understory Hymenoptera (Insecta) in Japanese cedar (Cryptomeria japonica D. Don) plantations. J Forest Res 12:14–23.
8. Nagai, M., Yoshida, T. 2006. Variation in understory structure and plant species diversity influenced by silvicultural treatments among 21- to 26-year-old Picea glehnii plantations. Journal of Forest Research, 11(1), 1-10.
9. Noguchi, M., Sakai, A., Okuda, S., Inagaki, Y., Fukata, H. 2009. Six-year responses of understory vegetation to thinning in hinoki cypress plantations in Shikoku Island, Japan. Jpn J For Environ 51:127–136.
10. Noguchi, M., Miyamoto, K., Okuda, S., Itou, T., Sakai, A. 2016. Heavy thinning in hinoki plantations in Shikoku (southwestern Japan) has limited effects on recruitment of seedlings of other tree species. Journal of Forest Research, 21:131. doi:10.1007/s10310-016-0522-9.
11. Nonoda, S., M. Shibuya, H. Saito, S. Ishibashi, and Takahashi, M. 2008. Invasion and growth processes of natural broadleaved trees and influences of thinning on the processes in an Abies sachalinensis plantation. Journal of the Japanese Forest Society 90:103–110.
12. Ohsawa, M. 2004. Species richness of Cerambycidae in larch plantations and natural broad-leaved forests of the central mountainous region of Japan. Forest Ecology and Management, 189(1-3), 375-385.
13. Ohsawa, M. 2005. Species richness and composition of Curculionidae (Coleoptera) in a conifer plantation, secondary forest, and old-growth forest in the central mountainous region of Japan. Ecological Research, 20(6), 632-645.
14. Ohsawa, M., Nagaike, T. 2007. Influence of forest types and effects of forestry activities on species richness and composition of Chrysomelidae in the central mountainous region of Japan. Biodiversity and Conservation, 15, 1179-1191.
15. Seiwa, K., Eto, Y., Hishita, M., Masaka, K. 2012. Effects of thinning intensity on species diversity and timber production in a conifer (Cryptomeria japonica) plantation in Japan. Journal of Forest Research, 17(6), 468-478.
16. Seiwa, K., Eto, Y., Hishita, M., Masaka, K. Imaji, A. Ueno, N., Hasegawa. Y., Konno, M., Kanno, H., Kimura, M. 2012. Roles of thinning intensity in hardwood recruitment and diversity in a conifer, Criptomeria japonica plantation: A 5-year demographic study. Forest Ecology and Management 269:177-187.
17. Shimada, H. Nonoda, T. 2009. Effects of Deer Browsing on Broad-leaved Tree Invasion after Heavy Thinning in Conifer Plantations. Journal of the Japanese Forest Society, 91(1) 46-50.
18. Shimada, H. Nonoda, T. 2009. Effects of evergreen shrubs on broad-leaved tree invasion for one year after canopy removal in Hinoki (Chamaecyparis obtusa) plantations. Journal of the Japanese Society of Revegetation Technology, 35 (1) 154-157.
19. Shoyama, K. 2013. Effects of thinning and excluding deer browsing on sapling establishment and growth in larch plantations. Landscape and Ecological Engineering, 9(1), 77-87.
20. Takasaki, Y., Takenaka, C., Yoshida, T. (2010). The Effect of Thinning on the Community Structure and Densities of Soil Animals in a Chamaecyparis obstusa Plantation. Journal of the Japanese Forest Society, 92(3), 167-170.
21. Taki, H., Inoue, T., Tanaka, H., Makihara, H., Sueyoshi, M., Isono, M., Okabe, K. 2010. Responses of community structure, diversity, and abundance of understory plants and insect assemblages to thinning in plantations. Forest Ecology and Management, 259(3), 607-613.
22. Tamura, A., Yamane, M. 2017. Response of understory vegetation over 10 years after thinning in an old-growth cedar and cypress plantation overgrazed by sika deer in eastern Japan. Forest Ecosystems, 4:1. DOI: 10.1186/s40663-016-0088-
23. Toyoshima, Y., Yamaura, Y., Yabuhara, Y., Nakamura, F. (2013). A preliminary study on the effects of line and selective thinning on bird communities in Hokkaido, northern Japan. Journal of Forestry Research, 24(3), 553-559.
24. Utsugi, E., Kanno, H., Ueno, N., Tomita, M., Saitoh, T., Kimura, M., Seiwa, K. 2006. Hardwood recruitment into conifer plantations in Japan: Effects of thinning and distance from neighboring hardwood forests. Forest Ecology and Management, 237(1-3), 15-28.
25. Watanabe, I., Yasaka, M., Koyama, H., Takiya, M., Ohno, Y. 2002. Diversity of the under-layer vegetation response to thinning in Todo fir (Abies sachalinensis) artificial forest. Transactions of the Meeting in Hokkaido Branch of the Japanese Forestry Society (50), 65-67.
26. Zhu, J. J., Gonda, Y., Yu, L. Z., Li, F. Q., Yan, Q. L., Sun, Y. R. 2012. Regeneration of a Coastal Pine (Pinus thunbergii Parl.) Forest 11 Years after Thinning, Niigata, Japan. Plos One, 7(10).

#### Information on study weighting

Following Mayerhofer et al. (2013), we estimated the relative weights as:

| $wt=(N_{C}N_{T})/(N_{C}+N_{T})$ , |  |
| --- | --- |

where $N_{C}$ and $N_{T}$ are the true sample sizes of the unthinned control and thinned treatments, respectively, identifying the number of spatially interspersed replicates of forest treatments (Halme et al. 2010).

The use of this weighting (multiplying the effect size by *wt*) down-weights the effect sizes with fewer total sample sizes (*N_c_* + *N_t_*). Because our meta-analysis could not incorporate within-study variance in observations, it potentially underestimates the meta-variance (Viechtbauer, 2016). We therefore interpret our results with a focus on effect size magnitude and direction, as opposed to precision and significance (Doncaster & Spake, 2017). In individual studies with low replication and low (near-zero) treatment values of richness or abundance, there is possibility of effect size overestimation (Lajeunesse, 2015). It was not possible to correct for this due to unreported or unavailable study variances (e.g. when *n* = 1).

**Table C.1.** Variables included in linear mixed models explaining variation in the log response ratio of sapling and seedling abundance of thinned to unthinned reference forest. In addition to the null model, only models with ∆AICc < 4 are shown, i.e. those with considerable support (Burnham and Anderson, 2002).

| Model | Fixed explanatory variables included in model | df | ∆AICc | AICc weight | Marginal R^2^ |
| --- | --- | --- | --- | --- | --- |
| 1 | time since + intensity + time since*intensity | 7 | 0.00 | 0.55 | 0.42 |
| 2 | time since + intensity + time since*intensity + stand age | 8 | 2.97 | 0.21 | 0.42 |
| Null |  | 4 | 10.89 | 0.003 | 0.00 |

**Table C.2.** Parameter estimates of the minimum adequate model explaining variation in sapling and seedling abundance response to thinning .

| Explanatory variable | Parameter estimate | Standard error | *P* |
| --- | --- | --- | --- |
| Intercept | -1.47 | 0.98 | 0.14 |
| intensity | 0.08 | 0.02 | <0.001 |
| time | 0.19 | 0.13 | 0.06 |
| intensity * time | -0.01 | 0.00 | <0.001 |

**Table C.3.** Variables included in linear mixed models explaining variation in the log response ratio of ground-layer plant abundance of thinned to unthinned reference forest. In addition to the null model, only models with ∆AICc < 4 are shown, i.e. those with considerable support (Burnham and Anderson, 2002).

| Model | Fixed explanatory variables included in model | df | ∆AICc | AICc weight | Marginal R^2^ |
| --- | --- | --- | --- | --- | --- |
| 1 | stand age + intensity | 6 | 0.00 | 0.19 | 0.25 |
| 2 | stand age + intensity + time since | 7 | 0.12 | 0.18 | 0.22 |
| 3 | intensity | 5 | 0.13 | 0.18 | 0.03 |
| 4 | stand age + intensity + stand age*intensity | 7 | 1.88 | 0.08 | 0.21 |
| 5 | stand age + intensity + time since + stand age*time since | 8 | 1.91 | 0.07 | 0.22 |
| 6 | time since + intensity | 6 | 2.04 | 0.07 | 0.06 |
| Null |  | 4 | 8.8 | 0.002 | 0.00 |

**Table C.4.** Full model-averaged parameter estimates and importance values for models of sapling and seedling abundance response to thinning with ∆AICc < 2, calculated by multiplying the estimates for individual models which contain parameters by their weights. Relative importance is the sum of the AICc weights across these models.

| Explanatory variable | Parameter estimate | Standard error | *P* | Importance value |
| --- | --- | --- | --- | --- |
| intercept | 0.00 | 0.00 | 0.27 |  |
| stand age | 0.02 | 0.01 | <0.001 | 0.85 |
| intensity | 0.01 | 0.00 | <0.001 | 0.85 |
| time since | 0.03 | 0.28 | 0.47 | 0.41 |

## Appendix D. Supporting information for the review of the effects of plantation rotation age extension on richness and abundance

#### Studies that measured biodiversity responses to plantation age


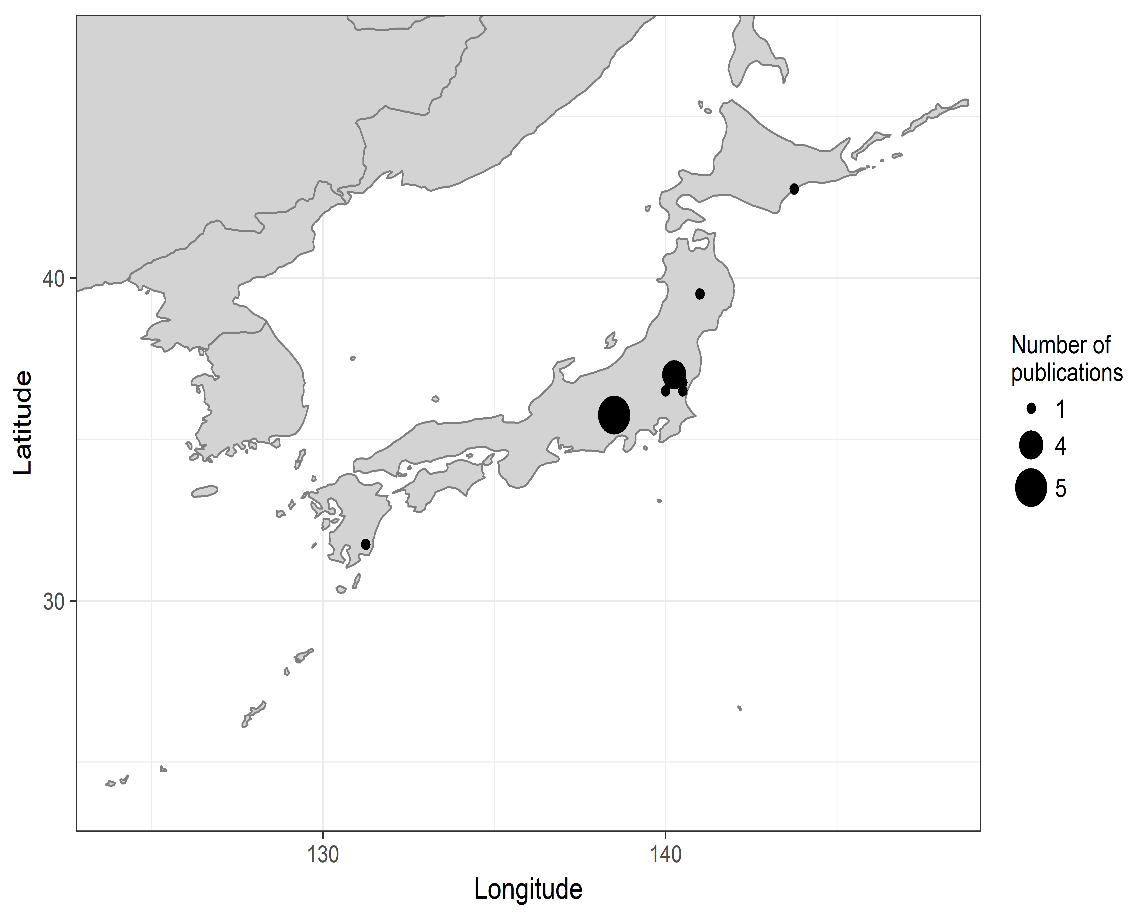


Figure D.1. Distribution of publications reporting biodiversity responses to plantation rotation age retrieved by this review.

1. Hasegawa, M., Fukuyama, K., Makino, S., Okochi, I., Goto, H., Mizoguchi, T., . . . Tanaka, H. 2006. Collembolan community dynamics during deciduous forests regeneration in Japan. Pedobiologia, 50(2), 117-126.
2. Hasegawa, M., Okabe, K., Fukuyama, K., Makino, S., Okochi, I., Tanaka, H., . . . Sakata, T. (2013). Community structures of Mesostigmata, Prostigmata and Oribatida in broad-leaved regeneration forests and conifer plantations of various ages. Experimental and Applied Acarology, 59(4), 391-408.
3. Igarashi, T., Masaki, T., Nagaike, T., & Tanaka, H. (2016). Species richness of the understory woody vegetation in Japanese cedar plantations declines with increasing number of rotations. Journal of Forest Research, 21(6), 291–299. https://doi.org/10.1007/s10310-016-0537-2
4. Ito, S., Nakagawa, M., Buckley, G. P., & Nogami, K. 2003. Species richness in sugi (Cryptomeria japonica D. DON) plantations in southeastern Kyushu, Japan: the effects of stand type and age on understory trees and shrubs. Journal of Forest Research, 8, 49-57.
5. Katoh, K., Yachi, M. (2002). Relationship among Vegetation Management, Plant Species Richness and Soil Chemistry in “Satoyama” Forest. Journal of The Japanese Institute of Landscape Architecture, 66(5), 521-524.
6. Makino, S., Goto, H., Hasegawa, M., Okabe, K., Tanaka, H., Inoue, T., & Okochi, I. 2007. Degradation of longicorn beetle (Coleoptera, Cerambycidae, Disteniidae) fauna caused by conversion from broad-leaved to man-made conifer stands of Cryptomeria japonica (Taxodiaceae) in central Japan. Ecological Research, 22(3), 372-381.
7. Nagaike, T., Hayashi, A., Abe, M., Arai, N. 2003. Differences in plant species diversity in Larix kaempferi plantations of different ages in central Japan. Forest Ecology and Management, 183, 177-193.
8. Ohsawa, M. 2005. Species richness and composition of Curculionidae (Coleoptera) in a conifer plantation, secondary forest, and old-growth forest in the central mountainous region of Japan. Ecological Research, 20(6), 632-645.
9. Ohsawa, M. 2006. Species richness of Cerambycidae in larch plantations and natural broad-leaved forests of the central mountainous region of Japan (vol 189, pg 375, 2004). Forest Ecology and Management, 229(1-3), 395-395.
10. Ohsawa, M., & Nagaike, T. 2006. Influence of forest types and effects of forestry activities on species richness and composition of Chrysomelidae in the central mountainous region of Japan. Biodiversity and Conservation, 15(4), 1179-1191.
11. Taki, H., Okochi, I., Okabe, K., Inoue, T., Goto, H., Matsumura, T., & Makino, S. 2013. Succession Influences Wild Bees in a Temperate Forest Landscape: The Value of Early Successional Stages in Naturally Regenerated and Planted Forests. Plos One, 8(2). doi: 10.1371/journal.pone.0056678
12. Taki, H., Yamaura, Y., Okochi, I., Inoue, T., Okabe, K., & Makino, S. 2010. Effects of reforestation age on moth assemblages in plantations and naturally regenerated forests. Insect Conservation and Diversity, 3(4), 257-265.
13. Toyoshima, Y., Yamaura, Y., Mitsuda, Y., Yabuhara, Y., & Nakamura, F. 2013. Reconciling wood production with bird conservation: A regional analysis using bird distribution models and forestry scenarios in Tokachi district, northern Japan. Forest Ecology and Management, 307, 54-62.
14. Yamashita, S., Hattori, T., & Tanaka, H. 2012. Changes in community structure of wood-inhabiting aphyllophoraceous fungi after clear-cutting in a cool temperate zone of Japan: Planted conifer forest versus broad-leaved secondary forest. Forest Ecology and Management, 283, 27-34.
15. Yamaura, Y., Royle, J. A., Shimada, N., Asanuma, S., Sato, T., Taki, H., & Makino, S. 2012. Biodiversity of man-made open habitats in an underused country: a class of multispecies abundance models for count data. Biodiversity and Conservation, 21(6), 1365-1380.

**Table D.1.** Variables included in linear mixed models explaining variation in the log response ratio of species richness of younger plantation forest to overmature planted reference forest. In addition to the null model, only models with ∆AICc < 4 are shown, i.e. those with considerable support (Burnham and Anderson, 2002).

| Model | Fixed explanatory variables included in model | df | ∆AICc | AICc weight | Marginal R^2^ |
| --- | --- | --- | --- | --- | --- |
| 1 | log_10_(stand age) + taxonomic group + log_10_(stand age)* taxonomic group | 20 | 0.00 | 0.99 | 0.54 |
| Null |  | 3 | 30.10 | 0.000 | 0.00 |

**Table D.2.** Variables included in linear mixed models explaining variation in the log response ratio of abundanceof younger plantation forest to overmature planted reference forest. In addition to the null model, only models with ∆AICc < 4 are shown, i.e. those with considerable support (Burnham and Anderson, 2002).

| Model | Fixed explanatory variables included in model | df | ∆AICc | AICc weight | Marginal R^2^ |
| --- | --- | --- | --- | --- | --- |
| 1 | log_10_(stand age) + taxonomic group + log_10_(stand age)* taxonomic group | 16 | 0.00 | 0.99 | 0.55 |
| Null |  | 3 | 16.65 | 0.000 | 0.00 |

## Appendix E. Supporting information for the review of the effects of traditional satoyama management of secondary forests on richness and abundance

#### Studies that measured biodiversity responses to traditional satoyama management


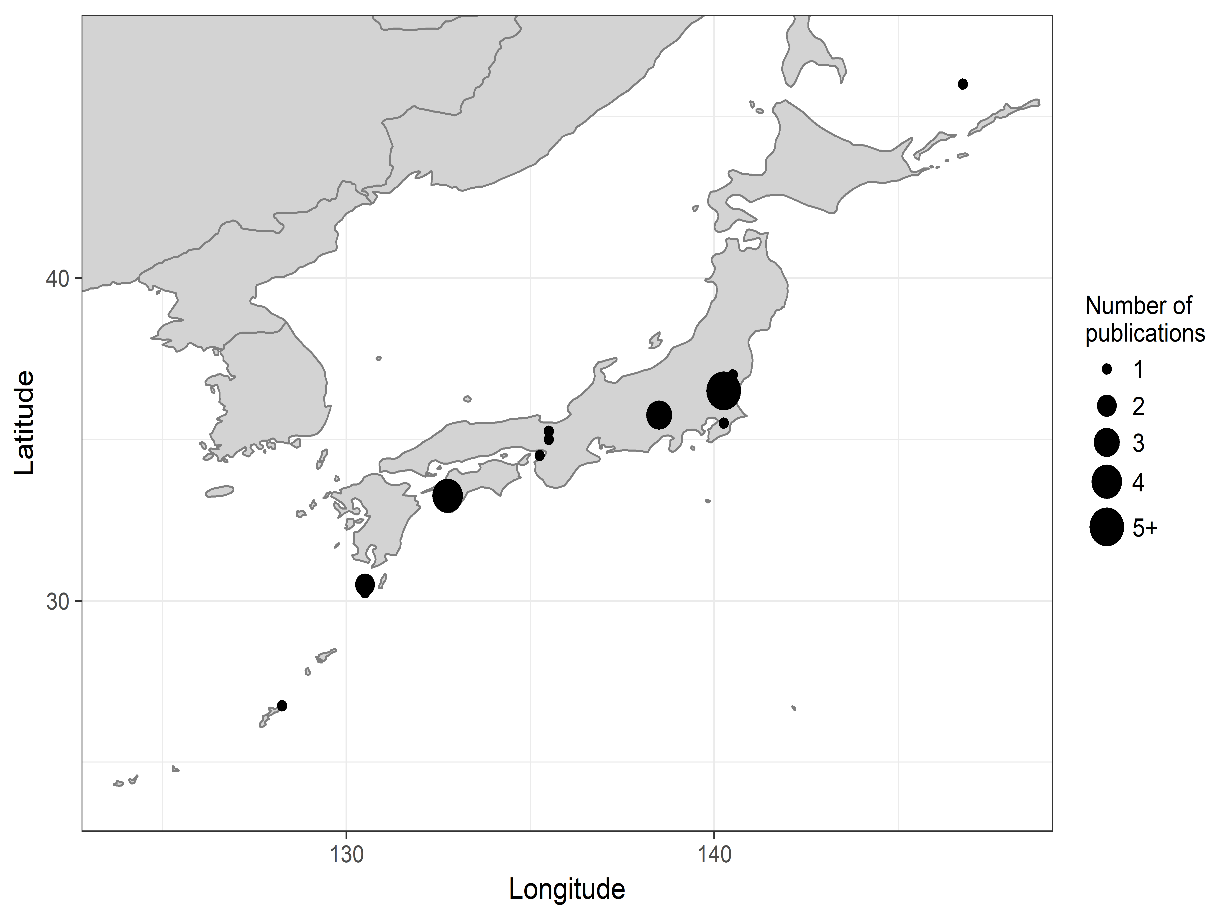


Figure E.1. Distribution of publications reporting biodiversity responses to traditional satoyama management retrieved by this review.

1. Aiba, S. I., Hill, D. A., & Agetsuma, N. (2001). Comparison between old-growth stands and secondary stands regenerating after clear-felling in warm-temperate forests of Yakushima, southern Japan. Forest Ecology and Management, 140(2–3), 163–175. https://doi.org/10.1016/S0378-1127(00)00325-X
2. Haraguchi, T. F., & Tayasu, I. (2016). Turnover of species and guilds in shrub spider communities in a 100-year postlogging forest chronosequence. Environmental Entomology, 45(1), 117–126. https://doi.org/10.1093/ee/nvv142
3. Hirayama, K., Yamada, S., Machida, H., & Yoshikawa, T. (2016). How do changes in forest stand development affect frugivorous bird abundance and fruit removal in warm-temperate forests of western Japan? Plant Ecology, 217(9), 1081–1094. https://doi.org/10.1007/s11258-016-0634-5
4. Hasegawa, M., Fukuyama, K., Makino, S., Okochi, I., Goto, H., Mizoguchi, T., . . . Tanaka, H. (2006). Collembolan community dynamics during deciduous forests regeneration in Japan. Pedobiologia, 50(2), 117-126.
5. Hasegawa, M., Fukuyama, K., Makino, S., Okochi, I., Tanaka, H., Okabe, K., Sakata, T. 2009. Collembolan community in broad-leaved forests and in conifer stands of Cryptomeria japonica in Central Japan. Pesquisa Agropecuaria Brasileira, 44(8), 881-890.
6. Hasegawa, M., Okabe, K., Fukuyama, K., Makino, S., Okochi, I., Tanaka, H., Sakata, T. 2013. Community structures of Mesostigmata, Prostigmata and Oribatida in broad-leaved regeneration forests and conifer plantations of various ages. Experimental and Applied Acarology, 59(4), 391-408.
7. Igarashi, T., Masaki, T., Nagaike, T., & Tanaka, H. (2016). Species richness of the understory woody vegetation in Japanese cedar plantations declines with increasing number of rotations. Journal of Forest Research, 21(6), 291–299. https://doi.org/10.1007/s10310-016-0537-2
8. Inoue, T. 2003. Chronosequential change in a butterfly community after clear-cutting of deciduous forests in a cool temperate region of central Japan. Entomological Science, 6, 151–163.
9. Ito, H., Hino, T., Sakuma, D. 2012. Species abundance in floor vegetation of managed coppice and abandoned forest. Forest Ecology and Management, 269, 99-105.
10. Kubota, Y., Katsuda, K., Kikuzawa, K. 2005. Secondary succession and effects of clear-logging on diversity in the subtropical forests on Okinawa Island, southern Japan. Biodiversity and Conservation, 14(4), 879-901.
11. Maeto, K., Makihara, H. 1999. Changes in insect assemblages with secondary succession of temperate deciduous forests after clear-cutting. Japanese Journal of Applied Entomology and Zoology, 2(1), 11-26.
12. Maeto, K., Sato, S. 2004. Impacts of forestry on ant species richness and composition in warm-temperate forests of Japan. Forest Ecology and Management, 187(2-3), 213-223.
13. Maeto, K., Sato, S. Miyata, H. (2002). Species diversity of longicorn beetles in humid warm-temperate forests: the impact of forest management practices on old-growth forest species in southwestern Japan. Biodiversity and Conservation, 11(11), 1919-1937.
14. Makino, S., Goto, H., Hasegawa, M., Okabe, K., Tanaka, H., Inoue, T., Okochi, I. 2007. Degradation of longicorn beetle (Coleoptera, Cerambycidae, Disteniidae) fauna caused by conversion from broad-leaved to man-made conifer stands of Cryptomeria japonica (Taxodiaceae) in central Japan. Ecological Research, 22(3), 372-381.
15. Makino, S. Goto, H. Inoue, T. Sueyoshi, M. Okabe, K., Hasegawa, M. Hamaguchi, K, Tanaka, H. Okochi, I. (2006). The monitoring of insects to maintain biodiversity in ogawa forest reserve. Environmental Monitoring and Assessment, 120: 477–485.
16. Maleque, M. A., Maeto, K., Makino, S., Goto, H., Tanaka, H., Hasegawa, M., Miyamoto, A. 2010. A chronosequence of understorey parasitic wasp assemblages in secondary broad-leaved forests in a Japanese 'satoyama' landscape. Insect Conservation and Diversity, 3(2), 143-151.
17. Ohsawa, M. 2004. Comparison of Elaterid biodiversity among larch plantations, secondary forests, and primary forests in the central mountainous region in Japan. Annals of the Entomological Society of America, 97(4), 770-774.
18. Ohsawa, M. 2004. Species richness of Cerambycidae in larch plantations and natural broad-leaved forests of the central mountainous region of Japan. Forest Ecology and Management, 189(1-3), 375-385.
19. Ohsawa, M. 2005. Species richness and composition of Curculionidae (Coleoptera) in a conifer plantation, secondary forest, and old-growth forest in the central mountainous region of Japan. Ecological Research, 20(6), 632-645. doi: 10.1007/s11284-005-0080-7
20. Taki, H., Okochi, I., Okabe, K., Inoue, T., Goto, H., Matsumura, T., Makino, S. 2013.
21. Sueyoshi, M., Maeto, K., Makihara, H. Makino, S, Iwai, T. (2003). Changes in dipteran assemblages with secondary succession of temperate deciduous forests following clear-cutting. Bulletin of FFPRI, 2(3).
22. Taki, H. Succession Influences Wild Bees in a Temperate Forest Landscape: The Value of Early Successional Stages in Naturally Regenerated and Planted Forests. Plos One, 8(2).
23. Taki, H., Yamaura, Y., Okochi, I., Inoue, T., Okabe, K., Makino, S. 2010. Effects of reforestation age on moth assemblages in plantations and naturally regenerated forests. Insect Conservation and Diversity, 3(4), 257-265.
24. Toyoshima, Y., Yamaura, Y., Mitsuda, Y., Yabuhara, Y., Nakamura, F. 2013. Reconciling wood production with bird conservation: A regional analysis using bird distribution models and forestry scenarios in Tokachi district, northern Japan. Forest Ecology and Management, 307, 54-62.
25. Yoshimura, M. 2009. Impact of secondary forest management on ant assemblage composition in the temperate region in Japan. Journal of Insect Conservation, 13(5), 563-568.

**Table E.1.** Variables included in linear mixed models explaining variation in the log response ratio of abundance of younger satoyama forest to >100-year-old reference forest. In addition to the null model, only models with ∆AICc < 4 are shown, i.e. those with considerable support (Burnham and Anderson, 2002).

| Model | Fixed explanatory variables included in model | df | ∆AICc | AICc weight | Marginal R^2^ |
| --- | --- | --- | --- | --- | --- |
| 1 | stand age + taxonomic group + stand age* taxonomic group | 20 | 0.00 | 0.99 | 0.35 |
| Null |  | 3 | 29.09 | 0.000 | 0.00 |

**Table E.2.** Variables included in linear mixed models explaining variation in the log response ratio of abundance of younger satoyama forest to >100-year-old reference forest. In addition to the null model, only models with ∆AICc < 4 are shown, i.e. those with considerable support (Burnham and Anderson, 2002).

| Model | Fixed explanatory variables included in model | df | ∆AICc | AICc weight | Marginal R^2^ |
| --- | --- | --- | --- | --- | --- |
| 1 | stand age + taxonomic group + stand age* taxonomic group | 12 | 0.00 | 0.87 | 0.38 |
| Null |  | 3 | 6.72 | 0.003 | 0.00 |

## Appendix F. Supporting information for the review of the effects of contemporary satoyama management of secondary forests on biodiversity

#### Studies that measured biodiversity responses to contemporary satoyama management

1. Abe, S., Kuramoto, S., Iida, S., S., S., Ishibashi, S., Takahashi, M., Masaki, T. 2013. Early responses of floor vegetation to the selection cutting in a mixed forest in Hokkaido, northern Japan. Journal of Japanese Forest Society, 95, 101–108. (in Japanese)
2. Azuma, S., Sasaki, T., and Ito, Y. 1997. Effects of undergrowth removal on the species diversity of insects in natural forests of Okinawa Hontokt. Pacific Conservation Biology, 3, 156-160.
3. Chikamatsu, M., Natuhara, Y., Mizutani, Y., Nakamura, A. 2002. Effect of artificial gaps on the butterfly assemblage in urban woods. Journal of the Japanese Society of Revegetation Technology, 28(1), 97-102. (in Japanese)
4. Hatase, Y., Oe, E., Oguri, H., Matsue, M., Utsugi, E., Imoto, I. 2006. Relationship between forest floor vegetation and forest management at the Michinoku Lakewood National Government Park in the Fagetea Crenatae region, Tohoku district. Landscape Research Japan Online, 69(5), 571-576. (in Japanese)
5. Hosogi, D., Kuno, H., Arai, K., Fukada, K. 2001. Effects of alteration of management to forest floor vegetation and environment of secondary coppice forests in urban area, Tokyo. I. Relation between the forest vegetation management method and growth of upper layer trees or forest floor vegetation. Journal of the Japanese Society of Revegetation Technology, 27(1), 14-19. (in Japanese)
6. Ito, Y., Aoki, J. 1999. Species diversity of soil-inhabiting oribatid mites in Yanbaru, the northern part of Okinawa Honto, and the effects of undergrowth removal on it. Pedobiologia, 43, 110-119.
7. Ito, Y., Takamine, H., and Yamauchi, K. 1998. Abundance and species diversity of ants in forests of Yanbaru, the northern part of Okinawa Honto with special reference to effects of undergrowth removal. Entomological Science, 1(3), 347-355.
8. Kanamori, T., Hashimoto, H., Yokouchi, S. 2009. Differences in the vegetation structure and their future succession between managed satoyama forests and abandoned ones in Suwa-cho, Tajimi City, Gifu Prefecture. *Meijyou Daigaku Nougaku-hou* (Agricultural Report of Meijyou University), 45, 5-16. (in Japanese)
9. Kato, K., Yachi, M. 2003. Relationship among vegetation management, plant species richness and soil chemistry in "Satoyama" forest. Landscape Research Japan Online, 66(5), 521-524. (in Japanese)
10. Kinoshita, A., Fukuda, H. 2004. Defference of fruting bodies of higher fungi between the sites with and without understory management. Japanese Journal of Forest Environment, 46(1), 29-34. (in Japanese)
11. Makino, A., Morimot, J., Shibata, S., Osawa, N., Nakanishi, A. 2002. *Toshi kinnkou niji-rinn ni okeru syou-mennseki bassai cyokugo no mokuhonn syokusei no hennka* (Change in diversity of woody vegetation just after tree cutting of small area in suburban secondary forests). Journal of the Japanese Society of Revegetation Technology, 28(1), 286-289. (in Japanese)
12. Matsumoto, K. 2012. Ground beetle assemblages in the forests of Tobuki-kita conservation area with special reference to effects of the understory management. Japanese Journal of Environmental Entomology and Zoology, 23(1), 9-17. (in Japanese)
13. Morito, J., Osawa, S., Katsuno, T. 2003. The relationship between species characteristics of woody seedling and the forest floor management by public participation in Satoyama parks. Journal of the Japanese Society of Revegetation Technology, 29(1), 239-242. (in Japanese)
14. Omine, T., Ito, Y. 1998. Abundance and diversity of soil macrofauna of forests in Yanbaru, northern montane part of Okinawa island, with special reference to removal of undergrowth. *Okinawa Daigaku Kiyo* (Report of Okinawa University), 15, 131-159. Available at: http://okinawa-repo.lib.u-ryukyu.ac.jp/handle/okinawa/5844
15. Sato, R., Aizawa, M., Kubota, K., Shibuya, S., Ohkubo, T. 2014. Carabid beetle assemblages in secondary deciduous broad-leaved forests with or without litter removal in northern Kanto. Journal of the Japanese Forest Society. 96(3), 141-145. (in Japanese)
16. Shibuya, S., Kubota, K., Kikvidze, Z., Ohsawa, M. (2008) Differential sensitivity of ground beetles, Eusilpha Japonica and Carabidae, to vegetation disturbance in an abandoned coppice forest in central Japan. Eurasian Journal of Forest Research, 11(2), 61-72.
17. Shibuya, S., Kubota, K., Ohsawa, M. 2008. Effects of small-scale management on biodiversity of an abandoned coppice forest in Japan: a case study on vegetation regeneration and ground beetle community. Web Ecology, 8, 116–124.
18. Shibuya, S., Kikvidze Z., Toki W., Kanazawa, Y., Suizu, T., Yajima, T., Fujimori, T., Mansournia, MT., Sule, Z., Kubota, K., Fukuda, K. 2014. Ground beetle community in suburban Satoyama – A case study on wing type and body size under small scale management. Journal of Asia-Pacific Entomology, 17, 775-780.
19. Shiota, M., Nakamura, A., Matue, N. 2004. Species diversity of woody seedling in planted laurel forest after management in an urban area and a secondary forest in suburbs. Journal of the Japanese Society of Revegetation Technology, 30(1), 116-120. (in Japanese)
20. Sueyoshi, M., Sato, H. 2010. Effect of the improvement cutting of natural forests on predacious flies (Diptera) in the Yambaru, a subtropical forest of Japan. Journal of the Japanese Forest Society, 92(5), 255-260. (in Japanese)
21. Taniwaki, T., Kuno, H., Kishi, Y. 2005. Comparison of ground beetle fauna on managed and short and long-term unmanaged floors in a suburban forest. Journal of the Japanese Society of Revegetation Technology, 31(2), 260-268. (in Japanese)
22. Suzuki, M. 2013 Succession of abandoned coppice woodlands weakens tolerance of ground-layer vegetation to ungulate herbivory: A test involving a field experiment. Forest Ecology and Management, 289, 318-324.
23. Yamamoto, T., Nakagoshi, N., Touyama, Y. 2001. Ecological study of pseudoscorpion fauna in the soil organic layer in managed and abandoned secondary forests. Ecological Research, 16, 593-601.
24. Yamasaki, H., Aoki, K., Hattori, T., Takeda, Y. 2000. Increase of species diversity due to management of vegetation in Satoyama (rural forest). Journal of the Japanese Institute of Landscape Architecture, 63(5), 481-484.
25. Yamase, K. 1998. Vegetational change with the magnitude of improvement cut in secondary forest of *Pinus densiflora*. Journal of the Japanese Institute of Landscape Architecture, 61(5), 567-570. (in Japanese)
26. Yamase, K. 2000. Kotonaru bassai kyoudo-ka deno syutugenn syokubutu no seiiku kannkyou kubunn to syusi sannpu yousiki (Growth environment and seed dispersal form of recruitment under different thinning intensities). Journal of the Japanese Society of Revegetation Technology, 25(4), 547-550. (in Japanese)
27. Yamase, K. 2008. Effect of the cover of evergreen broadleaved species on stand structure of coppice forest after the vegetation management. Landscape Research Japan Online, 71(5), 535-538. (in Japanese)
28. Yamase, K. Hattori, T., Mikami, K., Tanaka, A. 2005. Species richness and species composition of the coppice forests after “Hyogo-model” vegetation management. Landscape Research Japan Online, 68(5), 655-658. (in Japanese).

Table F1 Summaries of studies that investigated the impact of contemporary satoyama management on biodiversity.

| **Study** | **L^#^** | **M*** | **Study design and management details** | **Management objectives and actors** | **Taxonomic group and sampling method** | **Findings** | **Prefecture** |
| --- | --- | --- | --- | --- | --- | --- | --- |
| ***Fungi*** | | | | | | | |
| Kinoshita & Fukuda (2004) | J | TT,  LR | Selected a pair of managed and unmanaged coppice stands dominated by Pinus densiflora and Quercus serrata. Established a 10×10-m plot at each stand. Managed by improvement cutting and removing fallen or dead trees. | Maintaining *Pinus densiflora*- or *Quercus serrata*- dominated forests | Fruiting bodies of higher fungi | Species richness of ectomycorrhizal fungi and the percentage of roots colonised by ectomycorrhizae were higher in the unmanaged plot than in the managed plot. | Aichi |
| ***Soil invertebrates*** | | | | | | | |
| Yamamoto et al. (2001) | E | TT, UR | Selected five sites, each containing a pair of *Pinus densiflora*-dominated secondary forest stands: one abandoned and one managed. | Recreation, maintaining aesthetic appearance and for production of matsutake mushrooms | Pseudoscorpions sampled by hand sorting from the soil organic layer within a fixed circular (20-cm diameter) area. Five to eight samples were taken at each stand. | Pseudoscorpion species richness and densities were higher in abandoned stands than in managed stands. | Hiroshima |
| Omine & Ito (1998) | E | UR | Selected three sites, each containing a pair of managed and unmanaged forest stands dominated by *Castanopsis siieboldii*. Two, 50×50-cm quadrats established within each stand. | Government subsidised management as part of the “Natural Forest Improvement Project” | Soil macrofauna (myriapoda and diplopoda) sampled by hand sorting soil and litter within each quadrat. | Little difference in species diversity metrics between managed and unmanaged sites. | Okinawa |
| Ito & Aoki (1999) | E | UR | As Omine & Ito (1998) | As Omine & Ito (1998) | Orbatid mites sampled by Tullgren extraction | Species diversity was higher in unmanaged stands. | Okinawa |
| ***Insects*** | | | | | | | |
| Azuma et al. (1997) | E | UR | As Omine & Ito (1998) | As Omine & Ito (1998) | Insects sampled by sweeping method. | Species diversity was higher in unmanaged stands than managed stands, but the opposite was true for abundance. | Okinawa |
| Ito et al. (1998) | E | UR | As Omine & Ito (1998) | As Omine & Ito (1998) | Ants sampled by 30-minute hand searches in unknown plot sizes. | Species diversity was lower in managed than unmanaged forests, while abundance was higher in unmanaged stands. | Okinawa |
| Chikamatsu et al. (2002) | J | TT | Selected six evergreen broad-leaved stands with 15×15-m artificial gap thinned with high intensity (76~100% of stand volume). Established a pair of 15×15-m plots 16 m apart in forest interior and gap of each stand. | Biodiversity conservation | Butterflies recorded for 10 minutes in each stand. | Species richness and abundance was higher in thinned plots. | Osaka |
| Matsumoto (2012) | J | UR | Established 12 plots in a *Quercus serrata*–dominated secondary forest stands: six unmanaged and six managed by cutting shrub. | Biodiversity conservation by volunteers | Ground beetles, sampled with pitfall traps. | Species richness per plot did not differ detectably, but rarefaction curves for pooled samples suggested higher species richness in managed. No clear difference in abundance and the Simpson index of diversity. | Tokyo |
| Sato et al. (2014) | J | LR | Selected six, 30-40 year-old *Quercus*-dominated stands: three in abandoned stands and three in stands managed by litter removal. Established a 0.1-0.2-ha plot in each . | For making compost by the recycling centre for organic matter of the town | Ground beetles, sampled with pitfall traps | No clear differences in the abundance, species richness, and Simpson index of diversity were found between the forest floor treatments. | Tochigi |
| Shibuya et al. (2008a); Shibuya et al. (2008b) | E | TT, UR, LR | Set up five experimental plots within a 100×80-m *Quercus serrata*-dominated forest stand abandoned since 40 years. Different combinations of manipulations were applied to small (10×20-m) adjacent plots: i) all trees >1.3 m removed, ii) undergrowth removal, iii) litter removal. One plot was left unmanaged. | By researchers | Ground beetles, sampled with pitfall traps over three years. | Tree cutting increased the diversity of ground beetle species, while litter removal reduced their abundance.  *Eusilpha japonica*, considered an indicator species, had lower abundance in managed stands. | Saitama |
| Shibuya et al. (2014) | E | TT | Before-After-Control-Impact design. Established two adjacent 50×50-m plots in a *Quercus acutissima-*dominated stand. Removed 30% basal area in one plot. Measured biodiversity before and after thinning in both plots. | By researchers | Ground beetles, sampled with pitfall traps | Little compositional difference in thinned and unthinned plots. Abundance was higher in unthinned plots. | Chiba |
| Sueyoshi & Sato (2010) | J | TT | Established 16, 10×10 m plots in *Castanopsis sieboldii*-dominated stands: three unmanaged and 13 subjected to improvement cutting in previous years. | Improvement cut by the village or prefectural government. | Predacious flies sampled with emergence traps containing coarse woody debris | Abundance was higher in improvement cut plots and family composition changed. However, the effects on abundance and family composition continued for four and seven years, respectively. | Okinawa |
| Taniwaki et al. (2005) | J | UR, LR | Selected four 70-year-old *Quercus serrata*-dominated stands: one long-abandoned (since 27 years) stand, one recently abandoned stand (since 7 years) and two that were managed each year by undergrowth clearance and litter removal. Established a 20×20-m plot in each. | For compost production | Ground beetles, sampled with pitfall traps | Communities were similar in recently abandoned and managed forests, but differed to long-abandoned forests. The abundance of *Eusilpha japonica* was highest in long-abandoned sites. | Kanagawa |
| ***Plants*** | | | | | | | |
| Abe et al. (2013) | J | TT | Established two adjacent 1-ha plots in a mixed forest stand. Removed 20% basal area in one plot. Measured biodiversity in 25 or 24, 2×2-m quadrats in each plot in the following five years after cutting. | By researchers | Ground-layer vascular plants <2-m in height | Dwarf bamboo *(Sasa senanensis)* grew thickly in the cut plot. No clear difference in species richness or Shannon’s index were found between the cutting stand and the unmanaged stand. | Hokkaido |
| Hatase et al. (2006) | J | TT, UR | Selected 18 *Quercus serrata*-dominated stands: six unmanaged, six managed by undergrowth removal only and sixmanaged by thinning and undergrowth removal. Established a 10×10-m plot in each. | For research, managed by the park | Ground-layer herbaceous plants <0.5-m in height | Species richness was higher in managed than unmanaged stands. | Miyagi |
| Hosogi et al. (2001) | J | UR,LR | Selected two pairs of managed and unmanaged stands: *Quercus*-dominated stands (pair A) and *Carpinus laxiflora*-dominated stands (pair B). Unmanaged plot in stand A was abandoned 25 years ago, and that in stand B was abandoned five years ago. In managed plots, understory and litter removal have been conducted annually since over 50 years. A 20×20-m plot was established in each stand. | For compost and fertiliser production | Trees >2-m in height, and ground-layer plants estimated by the Braun-Blanquet method. | In pair B, species richness and cover of ground-layer perennial herbs was lower, and those of trees was higher in unmanaged stand. In pair A, species richness and cover of ground-layer plants was lower in unmanaged plot, while abundance and leaf area index of trees showed the contrasting pattern. | Tokyo |
| Kanamori et al. (2009) | J | UR,  TT | Established four, 20×20-m plots in stands dominated by *Quercus variabilis* and *Quercus serrata*: two abandoned and two managed by removing understory and evergreen broad-leaved trees 14 times in a year. | By local communities | Vegetation in multiple canopy layers | Species diversity index in the mid-canopy was higher in abandoned plots. | Gifu |
| Kato & Yachi (2003) | J | TT, UR, LR | Established line transects in 24, *Quercus serrata*-dominated secondary forest stands, abandoned for 1-30 years. Five, 2×2-m quadrats were spaced along each transect. | Managed by landowners | Trees whose height was more than 1.5 m in each transect, and Ground-layer plants <1.5-m in height in five quadrats along each transect. | As abandoned year was longer, species richness of woody plants increased, but species richness of ground-layer plants decreased. These relationships were clear in deciduous broad-leaved secondary forests, and were not found in conifer plantation forests. | Tochigi |
| Makino et al. (2002) | J | TT | Conducted retention forestry in *Chamaecyparis obtusa*-dominated s (0.21 ha), and divided it into three sections (30×30-m, 30×20-m, 15×40-m), containing one, 20×20-m or 15×25-m plot for sampling epicormic branches, and 36, or 24, 1×1-m plots for seedlings. | By researchers | Trees and seedlings | Species richness increased after harvesting. | Kyoto |
| Morito et al. (2003) | J | TT, UR, LR | Selected two study sites, each containing a pair of *Quercus* -dominated secondary forest stands: one managed and one unmanaged. Established two, 10×10-m plots in each stand. | For recreation, coppiced by community volunteers | Seedlings < 1.5m in height | Proportion of evergreen tree abundance to deciduous tree was higher in unmanaged sites than managed sites. | Kanagawa |
| Shiota et al. (2004) | J | TT | Selected two sites: one evergreen broad-leaved plantation, and the other a *Quercus variabilis*-dominated secondary forest. Established 19, 100-m^2^ plots: three were left unmanaged in each site, and 13 were subjected to artificial gap creation, by removing 56-100% of the basal area within in 15×15-m patches. | By researchers | Seedlings of woody plants | Species richness and abundance increased by thinning, while intensity had no clear effects. | Osaka |
| Yamase (1998) | J | UR | Before-After-Control-Impact design. Two *Pinus densiflora*-dominated secondary forests study sites were selected, each containing three small (20×20-m) stands that were: i) unmanaged, ii) managed by understory removal only (trees <3-cm diameter); and iii) managed by thinning (25% of trees >3-cm in diameter removed) and understorey removal. Established a 10×10-m plot in each stand, and sampled biodiversity before and after management. | By researchers | Plants over six years | Thinning increased species richness. | Hyogo |
| Yamase (2000) | J | TT | Before-After-Control-Impact design. Selected four, 30×30-m *Pinus densiflora* stands managed by: i) clear cutting, ii) high intensity thinning (all trees <8-cm in diameter), iii) low intensity thinning (all trees <6-cm in diameter), iv) unmanaged. Established one 10×10-m plot in each stand, and five, 2×2- m subplots therein. Measured biodiversity before and after cutting. | For improving environmental functions by the prefectural government | Plants and seedlings | Species richness increased as intensity of cutting was higher. Especially, early successional species and anemochory or zoochory species increased. | Hyogo |
| Yamase (2008) | J | TT, UR | Before-After design. Three *Quercus*-dominated stands stands were selected, and a single 10×10-m plot established in each. Managed by cutting lucidophyllous plants and bamboo grasses. Measured plant cover before and every one or two year after cutting. | By the prefectural government, for establishing deciduous forests and improving biodiversity. | Plants | Cover of deciduous trees increased in 11 years. Evergreen trees dramatically decreased due to cutting, except in a site which had high abundance of evergreen trees before cutting. | Hyogo |
| Yamase et al. (2005) | J | UR | Before-After design.  Selected nine sites, each contacting one to three secondary forest stands, and established a 10×10-m plot in each stand (total plot number is 20). Managed by cutting lucidophyllous shrubs and bamboo grasses. Measured biodiversity before cutting and after cutting for 7-9 years. After first cutting, two plots were managed by annual cutting bamboo grass, and two plots were managed by cutting lucidophyllous shrubs after four or five years. | As part of the “Satoyama Management Project in Hyogo” for keeping forest tall and increasing species diversity | Plants | Species richness had much increased in the first five years after the management, except for some areas that species such as *Dicranopteris linearis* were dominant. | Hyogo |
| Yamasaki et al. (2000) | J | TT, UR | Selected nine sites, each containing one or two secondary forest stands, and established a 10×10-m plot in each stand (total plot number is 12). Managed by cutting lucidophyllous shrubs and bamboo grasses. Measured biodiversity before and after cutting for 1-3 years. | As Yamase et al. (2005) | Plants | Species richness increased after management. | Hyogo |

## Appendix G. Japanese translations of figure legends and embedded figures


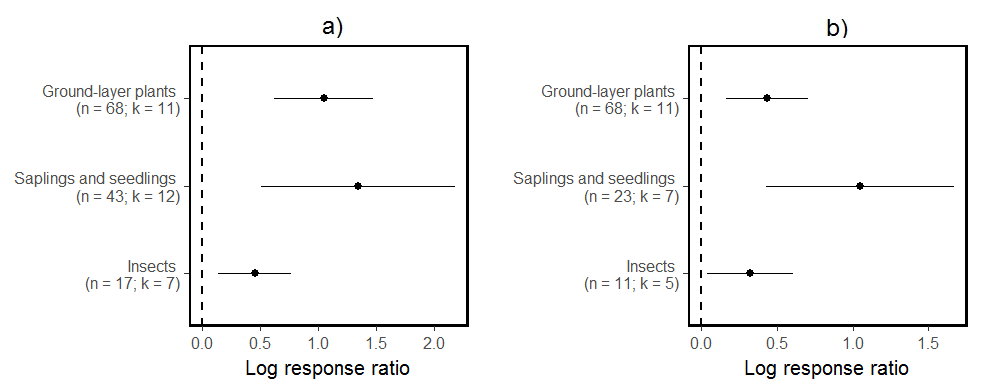


Figure 1. Summary mean effects and confidence intervals of thinning on a) abundance and b) species richness of biotic communities, from *n* studies at *k* study sites. 間伐が生物群集の種数（a）と個体数（b）に及ぼす平均効果の概要。k 個の文献、n 個の研究から得られた結果。


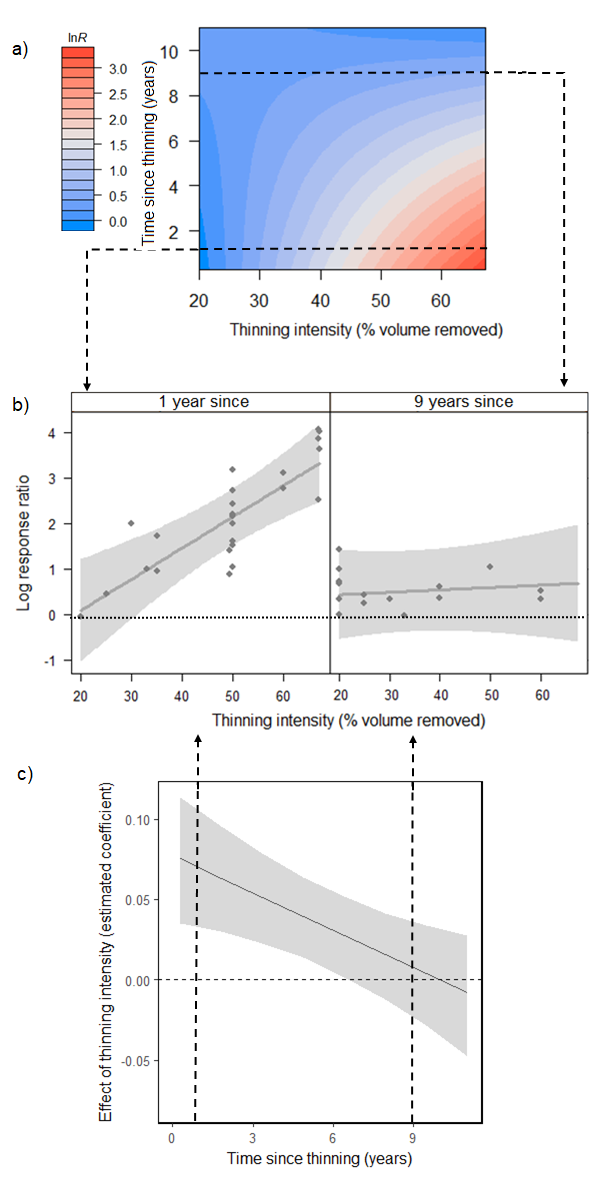


Figure 2. Impacts of plantation thinning on abundance of saplings and seedlings as dependent on time since thinning. a) Countour plot showing abundance differences (lnR) between thinned and unthinned forest stands as a function of thinning intensity and time since thinning. Dashed arrows point to sections through the plot at 1 and 9 years, illustrated in part b below. b) Influence of thinning intensity on abundance differences at 1 and 9 years since thinning, showing grey-shaded 95% CI in the regression based on between-study uncertainty in fixed effects only; values above horizontal dotted line signify higher abundance in thinned than unthinned stands. c) Marginal effect of thinning intensity, conditional on years since thinning; shading as for part b. Dashed arrows at 1 and 9 years show effects corresponding to response ratios in part b above. No effect of thinning intensity is detectable after six years. Regression used coefficients of the minimum adequate model.図2．人工林の間伐が実生・稚樹の個体数に及ぼす影響の間伐からの年数に応じた変化。（a）間伐からの経過年数（縦軸）と間伐強度（横軸：除去した樹木量の割合）が間伐の効果（間伐していない林分と比べた間伐した林分における個体数）に及ぼす影響を示した等高線プロット。色は間伐の効果の違いを表し、値が大きいほど間伐によって個体数が増加することを意味する。間伐の正の効果は、間伐強度が強く、間伐からの経過年数が短いほど高い（図中の赤色で示した領域）。（b）間伐から1年（左図）、9年（右図）が経過した林分における、間伐強度（横軸）が間伐の効果（縦軸）に及ぼす影響。水平な点線よりも上の林分では、間伐した林分における個体数が間伐していない林分に比べて多い。間伐から1年経過した林分では、間伐強度が強くなるほど間伐の効果が高くなる（推定された関係を示す実線が右肩上がり）が、9年経過した林分では間伐強度と間伐の効果の間に関係はみられない（実線が横ばい）。（c）間伐が個体数に及ぼす影響の間伐からの年数に応じた変化。横軸は間伐からの経過年数を、縦軸は間伐の影響（間伐強度に応じてどれだけ個体数が増加するか）を表す。間伐から年数が経過すると間伐の正の影響は小さくなり（推定された関係を示す実線が右肩下がり）、6年以上経過した林分では間伐の影響はみられなくなる。実線の描写には、説明力が高く、説明変数が少ないモデル（AIC最小モデル）を用いた。灰色の網掛け部は、固定効果（bでは間伐強度、cでは間伐からの年数）の推定誤差（メタ解析に使用した研究間でのデータのばらつき）に基づく95%予測区間を表す。


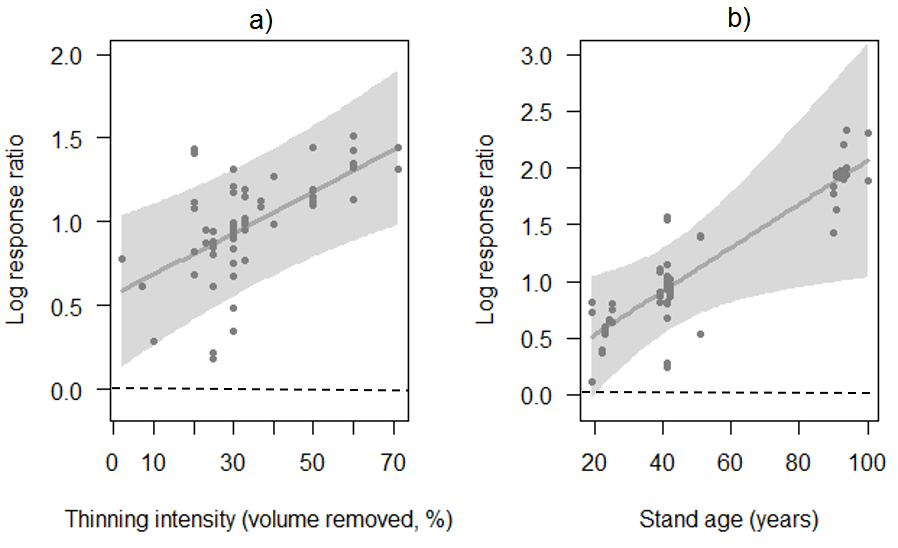


Figure 3. Influence of a) thinning intensity and b) stand age on abundance differences between thinned and unthinned plantation stands for understorey plants (horizontal dashed line means no difference). Regression used coefficients of the minimum adequate model. Grey shading shows 95% prediction intervals based on between-study uncertainty in fixed effects only. 間伐していない林分に対する間伐した林分における林床植物の個体数に間伐強度と林齢が及ぼす影響。水平な点線は間伐していない対照林分と間伐した処理林分の間に差がないことを表す。図の描写には、説明力が高く、説明変数が少ないモデル（AIC最小モデル）の係数を用いた。灰色の網掛け部は、固定効果の推定誤差に基づく95%予測区間（サンプリングしたデータの95％が含まれると予測される範囲）を表す。


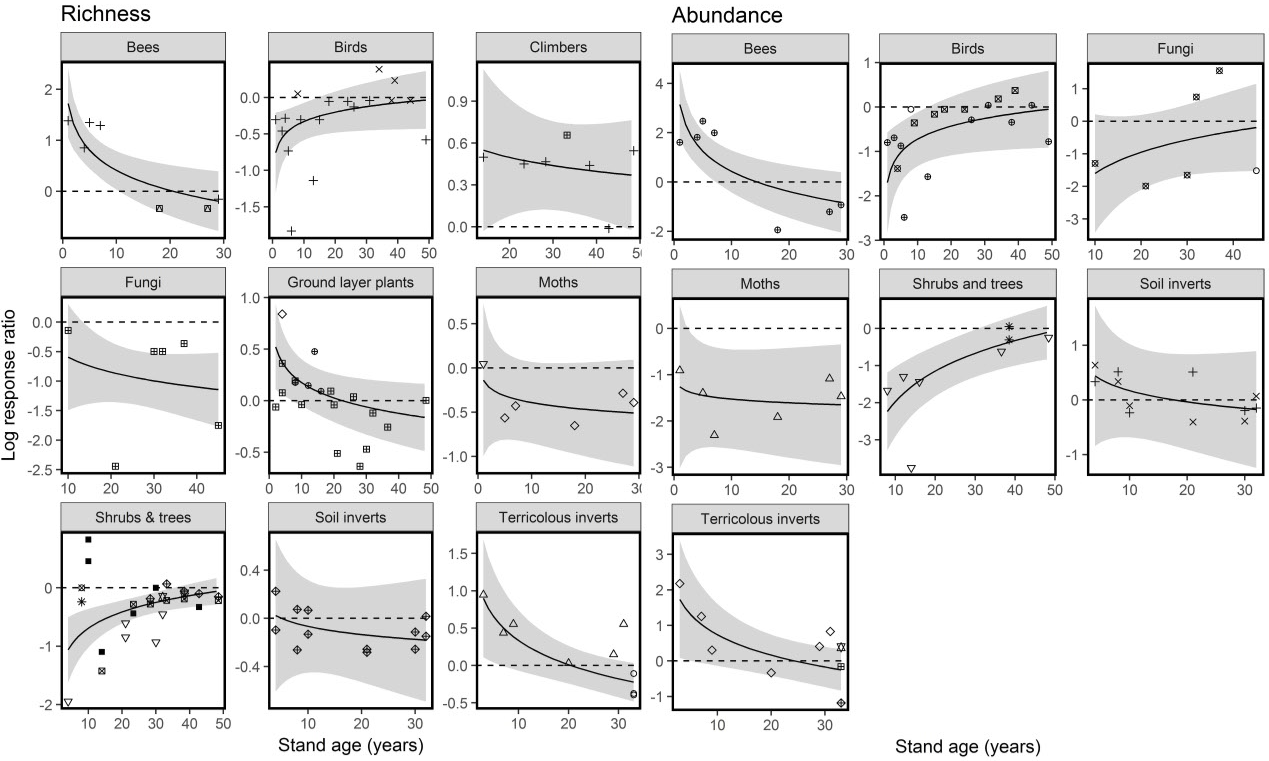


Figure 4. Influence of stand age on species richness and abundance effect sizes in planted stands relative to extended rotation planted stands. The horizontal dashed lines reference zero difference between extended rotation and younger treatment forest stands. Regressions used coefficients of the minimum adequate model. Grey shading shows 95% prediction intervals based on between-study uncertainty in fixed effects only. Different symbols correspond to different publications. 伐期を延長した老齢な人工林に対する若い人工林の林分における種数と個体数に林齢が及ぼす影響。水平な点線は、伐期を延長した老齢な対照林分とより若い処理林分の間に差がないことを表す。図の描写には、説明力が高く、説明変数が少ないモデル（AIC最小モデル）の係数を用いた。灰色の網掛け部は、固定効果の推定誤差に基づく95%予測区間（サンプリングしたデータの95％が含まれると予測される範囲）を表す。


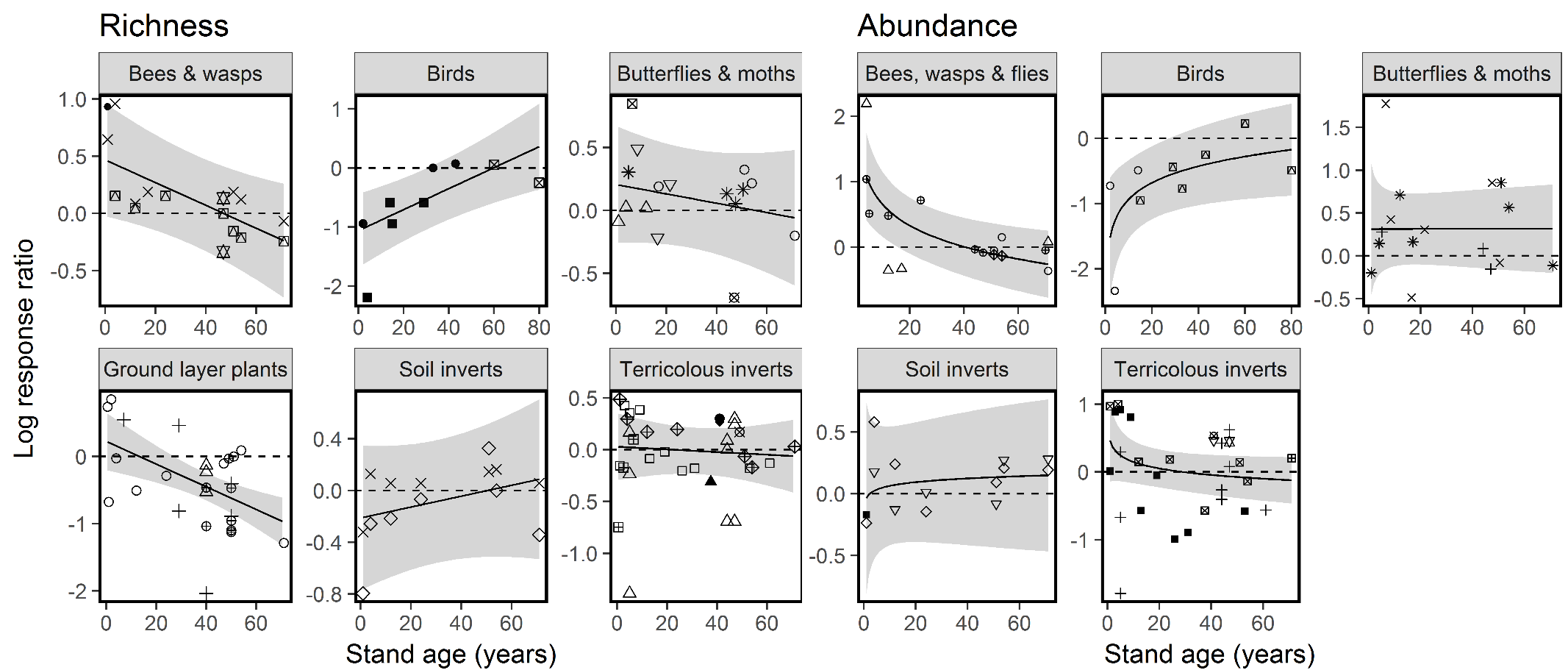


Figure 5. Influence of stand age on species richness effect sizes in younger secondary forest stands relative to older (>100-yr) abandoned forest stands. The horizontal dashed lines reference zero difference between extended rotation and younger treatment forest stands. Regressions used coefficients of the minimum adequate model. Grey shading shows 95% prediction intervals based on between-study uncertainty in fixed effects only. Different symbols correspond to different publications. 老齢な（林齢 > 100年）管理放棄された林分に対する若い二次林の林分における種数に林齢が及ぼす影響。水平な点線は、伐期を延長した老齢な対照林分とより若い処理林分の間に差がないことを表す。図の描写には、説明力が高く、説明変数が少ないモデル（AIC最小モデル）の係数を用いた。灰色の網掛け部は、固定効果の推定誤差に基づく95%予測区間（サンプリングしたデータの95％が含まれると予測される範囲）を表す。異なるシンボルは、異なる文献に対応して
